# Review on fiber composites for sustainable high strain rate applications

**DOI:** 10.1016/j.isci.2025.113598

**Published:** 2025-09-19

**Authors:** Darshan Madhapura Lakshme Gowda, Ravi Shankar Bhat, Sanjay Mavinkere Rangappa, Suchart Siengchin

**Affiliations:** 1 Department of Metallurgical and Materials Engineering, National Institute of Technology Karnataka, Surathkal, Manglore 575025, India; 2 Natural Composites Research Group Lab, Department of Materials and Production Engineering, The Sirindhorn International Thai-German Graduate School of Engineering (TGGS), King Mongkut’s University of Technology North Bangkok (KMUTNB), Bangkok, Thailand

**Keywords:** Composite materials, Materials science, Materials synthesis

## Abstract

Over the past two decades, the growing demand for sustainable, high-performance materials has driven significant advancements in fiber reinforced polymer composites (FRPCs), particularly for dynamic and ballistic applications. This review provides an integrated overview of recent developments, highlighting sustainable reinforcements, novel stacking configurations, and advanced machine learning (ML) predictive approaches. Particular emphasis is placed on bio-inspired helicoidal laminates and hybrid architectures, which offer superior energy absorption, damage tolerance, and impact mitigation. Hybrid laminates incorporating satin weaves, high-modulus fibers, and compliant matrices enhance post-impact toughness and better structural integrity. Additionally, embedding a high-hardness (70–90 Shore A) rubber core with a compliant matrix mitigates conical crack propagation, improves strain rate sensitivity, and reduces delamination under both low- and high-velocity impacts. The dynamic response of FRPCs is examined using experimental techniques such as Split Hopkinson Pressure Bar (SHPB) testing and impact assessments, revealing the influence of design variables on strain-rate-dependent behavior. To support material selection and design optimization in fiber composites, ML techniques, Ashby charts, and Multi-Criteria Decision-Making (MCDM) frameworks are explored, balancing performance, sustainability, and manufacturability. Failure mechanisms such as delamination, fiber pull-out, and matrix cracking are analyzed with respect to hybridization strategies and environmental effects. Finite element analysis (FEA) tools, including ABAQUS, ANSYS AUTODYN, and LS-DYNA, are reviewed for their predictive accuracy, validated against experimental results. Standardized testing protocols (ASTM D7136, D7137, F1342; NIJ-0101.06) ensure the consistent evaluation of both flexible and rigid armor systems. The review also discusses manufacturing advancements such as resin transfer molding (RTM) and filament winding, which improve scalability and reduce waste. Non-destructive testing (NDT) methods, including acoustic emission, ultrasonic C-scanning, and X-ray CT, are highlighted for real-time damage assessment. Finally, integrating ML algorithms such as MLP, SVM, RF, and CNN with experimental and simulation data enhances predictive modeling, damage classification, and tailored composite design. This convergence of bio-inspired design, computational tools, and intelligent systems is accelerating the development of next-generation FRPCs for aerospace, defense, automotive, and civil engineering applications.

## Graphical abstract

**Figure:**
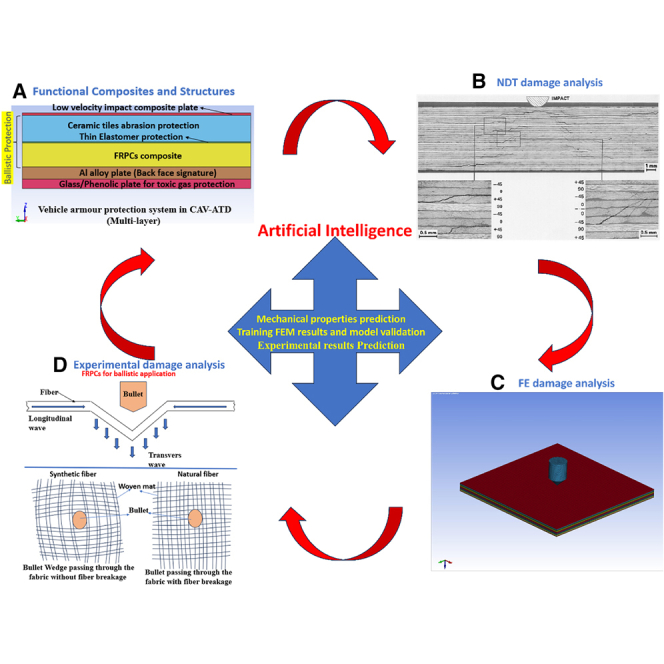

## Introduction

The growing societal concern for environmental sustainability has driven the development and adoption of recyclable and eco-friendly advanced materials.^1^ Fiber-reinforced polymer composites (FRPCs), composed of two or more distinct materials in intimate contact at the micro- and macroscopic scales, have emerged as versatile materials offering a wide range of applications.^2^^,^^3^ FRPCs find extensive applications in aerospace, military, sports, leisure, food packaging, transportation, civil construction, and maritime industries.^4^^,^^5^^,^^6^^,^^7^ With 31%, 26%, and 12% of the total FRPC market share dedicated to automotive, construction, and marine applications, respectively, impact resistance and durability are critical requirements. Between 2021 and 2028, the global FRPC market experienced a compound annual growth rate (CAGR) of 6.2%, increasing from $88.1 billion to $135.1 billion.^8^ Recent innovations in sustainable FRPCs include the replacement of carbon and glass fibers with natural flax fibers in surfboard manufacturing, which demonstrated performance comparable to synthetic fibers while offering better vibration-damping properties, thereby enhancing user experience.^9^ For dynamic impact applications, Correia et al.^10^ recommended the use of expanded cork as a lightweight alternative to oil-based foams, it reducing the composite’s weight by 62.8% comparatively and offers sustainability design.

FRPCs play a crucial role in boat masts and propeller design, which are under dynamic loading. The use of hybrid glass/carbon fiber composites enhances dynamic performance through fiber synergy. Compared to aluminum shafts, these composites achieve a weight reduction of 20–50%.^11^ Fiber materials are cost-effective and lightweight, which helps reduce production costs and fuel consumption, making them a preferred alternative for various applications.^12^ Most likely, FRPCs are widely used in various aspects of daily life. Therefore, it is essential to consider their current applications and future advancements.

**Figure 1:**
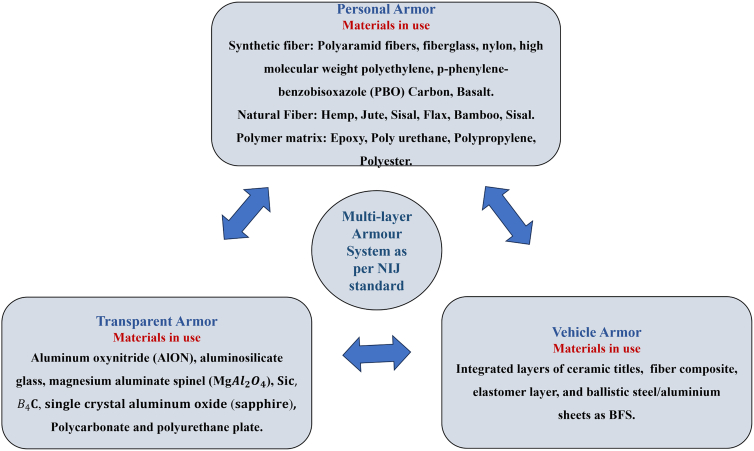
Classification of materials considered for light weight structure and multi-layer armor system (MAS)

**Figure 2:**
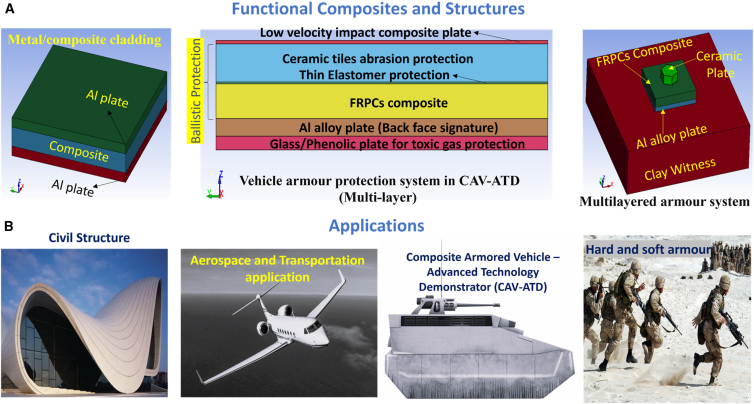
Functional composites and multi-layer armor system (MAS) applications (A) Functional composites and structures: schematic of metal/FRPC hybrid composites used for LVI and combat vehicle hard armor. (B) Lightweight protection material applications and typical MAS arrangements (ceramic front plate, FRPC core, elastomeric interlayer, metal backing). Adapted from Ref.^18^

The evolving demand for dynamic safety and protection across various engineering fields, particularly in military applications, have intensified with the advancement of sophisticated weaponry and the need for advanced armor design. Traditional monolithic materials, such as steel and ceramic-metal plates, are often heavy, expensive, and less suitable for mobility-focused armor systems.^13^^,^^14^ To overcome these challenges, advanced fiber composites, including para-aramid, ultra-high molecular weight polyethylene (UHMPE), glass, poly-benzobis-oxazole (PBO), M5 (polyhydroquinone-diimidazopyridine or PIPD), and carbon fibers, have increasingly replaced conventional materials, offering improved performance in lightweight, high-strength armor applications.^15^^,^^16^^,^^17^ Figure 1 illustrates the classification of materials selected for lightweight structures and MAS, along with the corresponding design requirements for various applications. A simplified MAS design typically comprises a sequence of distinct material layers, including a front ceramic plate, an FRPC core integrated with an elastomeric layer, and a ductile metal plate serving as the back-face signature (BFS), as illustrated in Figure 2A. According to the National Institute of Justice (NIJ) standards, the BFS deformation for Level IV hard armor must not exceed 44 mm.^19^ To achieve the functional requirement of design, a glass/phenolic laminate is integrated in chobham armor to protect from toxic gas emission and shield against high-temperature fragment particles in the subsequent bullet interaction. The ceramic front plate plays a crucial role in breaking the incoming projectile into fragments, absorbing a maximum portion of bullet kinetic energy, and facilitating shock wave impedance transmission to the softer FRPC core. Due to density variations between the ceramic plate and the FRPC core, partial tensile shock waves reflect at their interface, which intensifies ceramic fragmentation and enhances further energy dissipation of impact.^20^^,^^21^ Monteiro et al.^22^ reported that 10 mm thick Al_2_O_3_ ceramic plates in hard armor systems dissipate up to 71.75% of the total impact energy of 3.487 kJ from a 7.62 NATO bullet. Compared to Kevlar/Polyester laminate with an equivalent of 30 vol. % reinforcement, jute-reinforced polyester laminate exhibits a 7–9% higher energy dissipation capacity. These findings highlight the potential of bio-based FRPCs in developing sustainable and effective ballistic protection systems.^23^^,^^24^^,^^25^ The MAS for CAV-ATD vehicle, hard/soft armor, and light weight metal sheet cladded composites for different engineering applications are shown in Figures 2A and 2B.

The adoption of natural and hybrid fiber laminates as intermediate layers in MAS offers a sustainable alternative to synthetic fibers. These laminates demonstrate superior kinetic energy dissipation through mechanisms such as fiber decohesion, which is less pronounced in synthetic fibers.^26^ Bio-inspired structural designs, including cross-ply, unidirectional, bi-directional, quasistatic, 3D-mat, helicoidal, and other optimized stacking configurations, further enhance the impact resistance and damage tolerance of FRPCs.^27^^,^^28^^,^^29^ Experimental techniques such as Split Hopkinson Pressure Bar (SHPB), gas gun apparatus, and blast testing, combined with multi-scale finite element (FE) modeling approaches, validate the performance of these materials under dynamic loading conditions.^14^^,^^30^^,^^31^^,^^32^^,^^33^

**Figure 3:**
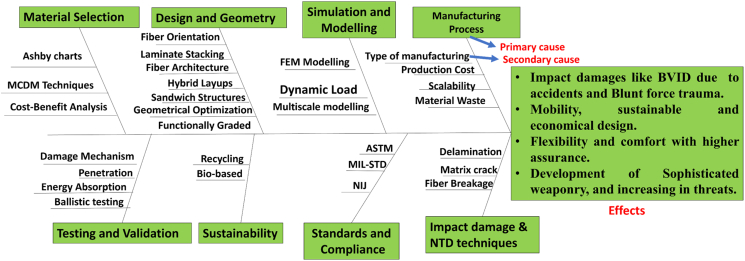
Ishikawa or cause-and-effect diagram for light-weight protection material development

Barely Visible Impact Damage (BVID) in composite materials is a critical concern in aerospace applications, where structural integrity and reliability are paramount. BVID often results from low-velocity impacts (LVI), such as tool drops or debris strikes, which may not cause visible surface damage but can induce internal failures such as matrix cracking, fiber breakage, and delamination.^34^^,^^35^ Traditional inspection techniques often fail to detect these hidden damages, necessitating advanced non-destructive testing (NDT) methods. Enhancing impact resistance through optimized stacking sequences and robust inspection methodologies is essential for improving composite durability and ensuring structural safety in real-world applications.^36^^,^^37^ Figure 3 demonstrates an Ishikawa diagram methodically addressing the causes of possible problems and illustrating practical solutions.^38^ The key factors influencing ballistic material performance and its characterization are material selection, design and geometry, simulation and modeling, manufacturing process, testing and validation, sustainability, standards and compliance, impact damages, and NDT techniques constitute the primary causes for the effect of BVID, blunt force trauma, revision in measured standards, and sophisticated development in weaponries.

**Figure 4:**
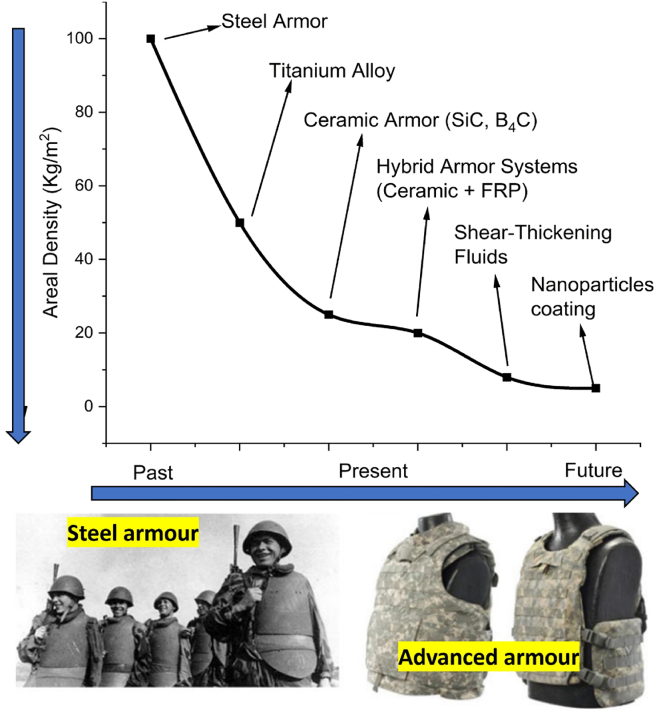
Areal density of armor Areal density comparison between conventional and advanced armor materials, showing reductions achieved through composite and hybrid configurations. Data adapted from Ref.^39^

The rapid evolution of lightweight ballistic materials has also emphasized sustainability and mobility as primary design considerations. FRPC-based lightweight structures not only improve mobility but also minimize operational fatigue. The transition from traditional rolled homogeneous steel to advanced armor materials, as illustrated in Figure 4, demonstrates significant reductions in areal density while enhancing performance.^13^^,^^40^ Continued advancements in material innovation, design optimization, and rigorous validation will enable next-generation ballistic protection systems that align with modern sustainability goals.^41^^,^^42^^,^^43^

**Figure 5:**
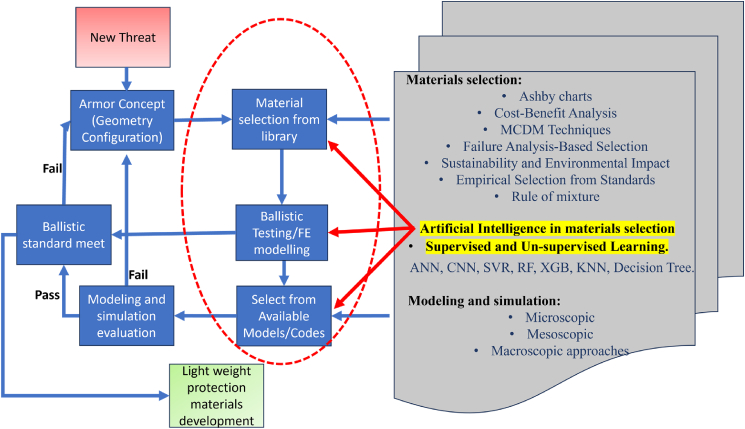
Revised methodology for new materials development for new threat causes with machine learning methods

The rapid evolution of sophisticated weaponry and upgraded threat levels necessitates continuous advancements in ballistic performance. Organizations such as the NIJ in the United States, the Standardization Agreement (STANAG) by the North Atlantic Treaty Organization (NATO), and the Association of Test Laboratories for Bullet-Resistant Materials and Constructions (VPAM) in Germany have implemented standardized guidelines to enhance armor designs with superior ballistic capabilities.^44^ Armor materials must exhibit higher impact resistance and damage tolerance to counter more potent weapons, explosives, and armor-piercing bullets. Moreover, the demand for materials capable of multi-threat resistance, offering protection against blast waves, shrapnel, and bullets, has increased significantly. In this context, researchers are actively exploring dynamic and adaptive materials that can modify their characteristics in response to varying threat levels, thus providing enhanced protection on demand.^1^^,^^29^ Meeting these challenges will require a holistic approach encompassing armor material optimization and selection, advanced modeling and simulation techniques, rigorous ballistic testing, and regular standards revision. Machine learning (ML) techniques utilizing both supervised and unsupervised learning play a crucial role in material selection, mechanical properties prediction for finite element analysis, and experimental result estimation.^45^^,^^46^ Integrated ML approaches are increasingly employed in developing advanced materials to address emerging challenges, as illustrated in Figure 5.

Comfort and flexibility are equally important considerations for personal protective equipment, as mobility is vital for wearers. Armor materials must strike a balance between stiffness for protection and flexibility for comfort. Ergonomically designed materials that fit the contours of vehicles or individuals improve both comfort and protection.^40^ Furthermore, breathable materials significantly reduce heat discomfort during prolonged usage. Advanced textiles and hybrid multilayer materials have been developed to meet these demands, providing enhanced comfort alongside robust protection.^18^^,^^47^

Another significant market requirement is cost-effective design. While delivering high performance, armor materials must remain economically viable for large-scale manufacturing.^48^ FRPCs are emerging as cost-effective alternatives to traditional materials such as steel and ceramics, particularly when incorporating hybrid blends of natural and synthetic fibers. Advances in automated production technologies, such as 3D printing and precision fiber placement, are reducing manufacturing costs without compromising quality.^49^^,^^50^^,^^51^ Scalability in production is essential for ensuring consistent quality and affordability. Moreover, durability and longevity are critical for maintaining continuous protection under extreme environmental conditions, minimizing replacements, and ensuring reliability over time.^52^^,^^53^^,^^54^

Machine learning (ML) techniques have revolutionized various engineering domains, enhancing damage detection, predictive maintenance, and process optimization. Neural networks, genetic algorithms, and reinforcement learning play a crucial role in adaptive control, particularly in complex systems.^55^ Bayesian optimization and decision trees further aid in parameter tuning and process monitoring. Advanced tools such as TensorFlow, PyTorch, and Scikit-Learn have streamlined ML applications, facilitating their integration into engineering research.^45^^,^^46^^,^^56^ The application of ML techniques for FRPCs in ballistic applications, including artificial neural networks (ANN), convolutional neural networks (CNN), and multilayer perceptrons (MLP), has significantly improved finite element analysis (FEA), predictive modeling, material functionalization, and failure mechanism analysis.^55^^,^^57^ These approaches offer enhanced accuracy and efficiency in material design and performance evaluation. However, their application in ballistic impact analysis remains limited, despite their potential to provide deeper insights into damage mechanisms, energy absorption, and impact resistance of fiber composites. Exploring ML-driven methodologies in ballistic applications could lead to optimized composite structures with superior protective capabilities.

Despite significant advancements in FRPC development for ballistic and dynamic applications, several technological gaps persist. The trade-off between impact resistance and material sustainability remains unresolved, especially for bio-composites with natural fibers, which often demonstrate inconsistent mechanical properties due to its variation in regional vegetation and its constituent characteristics. Lastly, the integration of bio-inspired designs into practical manufacturing workflows has not been fully realized, limiting scalability.

This review explores the state-of-the-art developments, challenges, and future prospects in FRPCs for dynamic and ballistic applications, with a focus on sustainable, high-performance materials for lightweight structures. While numerous stacking sequences and hybrid configurations have been studied, comparative benchmarking across loading regimes, threat levels, and material systems is limited. Progress in finite element analysis, manufacturing techniques, and material functionalization has enabled FRPCs to meet stringent performance criteria, particularly in ballistic defense systems where damage tolerance and energy dissipation are critical. Additionally, integrating machine learning techniques such as artificial neural networks (ANN), convolutional neural networks (CNN), and multilayer perceptron (MLP) has enhanced predictive modeling, finding the material properties, materials stacking, their optimization, and failure analysis with unprecedented accuracy. Machine learning (ML) applications in this domain are still in their infancy and lack standardization for predictive modeling, damage detection, and stacking optimization. The development of multi-layered armor systems (MAS) combining ductile plates, FRPCs, and ceramics highlights their role in improving mobility, reducing weight, and ensuring cost efficiency. While synthetic fibers such as aramid and UHMWPE dominate ballistic applications, ongoing research into natural fibers and bio-based composites offers a promising path toward sustainable yet high-performance materials. Future research must adopt multidisciplinary approaches to enhance impact resistance, mitigate Barely Visible Impact Damage (BVID), and leverage data-driven techniques for mechanical properties prediction and design optimization, reinforcing FRPCs as next-generation materials for evolving engineering, environmental, and societal challenges. The structure of this review is as follows: Section 2 discusses material selection strategies using design methodologies such as Ashby charts and MCDM methods for optimized performance. Section 3 highlights the influence of stacking sequences and geometries in FRPCs under dynamic loading. Section 4 focuses on finite element modeling for simulating ballistic and high strain rate responses. Section 5 reviews fabrication techniques and their impact on performance. Section 6 presents experimental validation methods across various strain rate regimes. Section 7 elaborates on ASTM standards relevant to ballistic evaluation. Section 8 covers non-destructive testing techniques for assessing impact damage. Section 9 explores the application of artificial intelligence in predicting and optimizing FRPC behavior under ballistic conditions.

## Literature on materials selection methods

For effective material selection in lightweight protection and MAS, this section explores conceptual design methodologies such as the Ashby chart for material screening.

**Figure 6:**
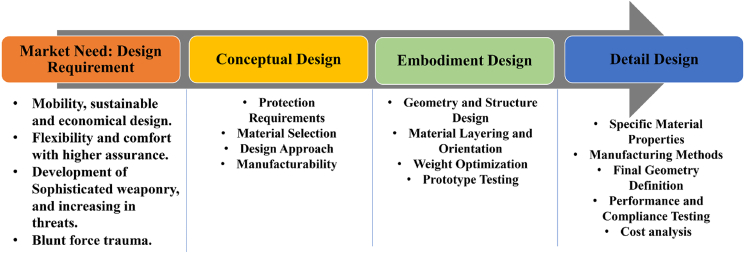
Product development process in light weight protection and MAS

The screening process is guided by problem-defining criteria identified from the literature. Figure 6 illustrates the design phases involved in the development of lightweight protection materials, highlighting a systematic approach to achieving optimal material performance.

### Case study on conceptual materials selection for armor design and problem defining criteria (problem defining criteria)

During dynamic impact, both longitudinal and transverse shock waves are induced as a result of the sudden load application. These shock waves cause material movement in the out-of-plane direction.^58^ The velocity of the longitudinal sound wave in a solid, referred to as sonic velocity, is related to the dynamic modulus of elasticity (E) and the material’s mass density (ρ), as given by the Equation 1.^59^

(Equation 1) $$C_{i}=\sqrt{\frac{E}{\rho }}$$

Where “C_i_” is the longitudinal wave velocity. Monolithic rolled homogeneous steel has traditionally been used in military chobham and body armor design. However, its use has become limited due to design constraints such as areal density, specific strength-to-weight ratio, energy absorption capabilities, and cost.

For initial material screening in lightweight ballistic designs, monolithic steel properties are considered. The longitudinal wave velocity in steel is approximately 5960 m/s. By applying the logarithm to both sides of the equation and assigning a constant “C_i_,” we obtain the Equation 2.

(Equation 2) Log(E)=Log(ρ)+7.550

**Figure 7:**
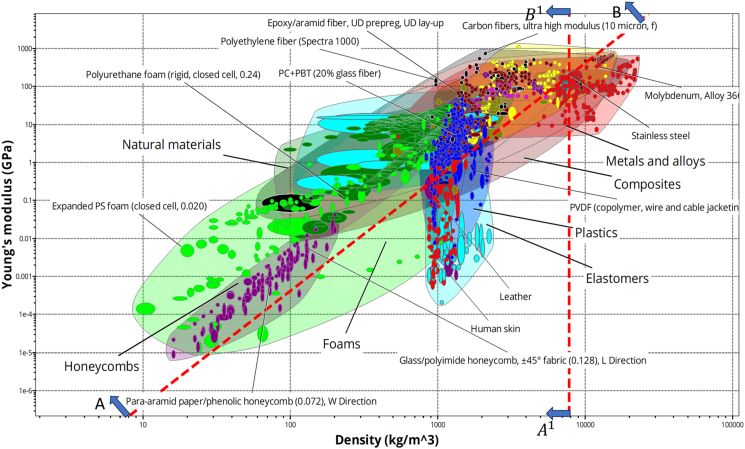
Ashby chart on Elastic modulus vs. Density for material selection Ashby chart illustrating the relationship between elastic modulus and density to identify suitable lightweight materials for armor applications. Figure adapted from Ref.^60^

This resembles a straight-line equation with a slope of 1 and a constant y-intercept of 7.55. Using the Ashby chart and considering steel properties such as Young’s modulus, density, and the guideline “AB” and “A^1^B^1^,” materials were selected for minimizing weight as the primary design criterion. Figure 7 highlights that the advanced composites, fiber-reinforced polymer composites (FRPCs), ceramics, and lightweight alloys are more suitable materials for armor design based on the weight reduction criterion.

Fracture in dynamic impact events typically occurs through the formation of multiple cracks. To design armor for high energy absorption, materials should be able to absorb elastic strain before fracture. This requires high toughness to resist back deflection elastically before it deforms permanently. The stress at which fracture is mitigated in materials after impact is given by Equation 3.^59^

(Equation 3) $$\sigma_{f}=\frac{GK_{1G}}{\sqrt{\pi a_{G}}}$$

This fracture stress condition is critical for both energy-limited and deflection-limited design approaches. Where “K_1G_” is the fracture toughness, “a_G_” is the crack length after fracture, and “G” is the geometry constant. Furthermore, the maximum elastic energy per unit volume (*U*_*e*_) , stored in the material, is given by the integral over the stress-strain curve up to the elastic limit as given in Equation 4.

(Equation 4) $$U_{e}=\frac{\sigma \times \epsilon }{2}=\frac{\sigma^{2}}{2E}$$

Impact failure due to fracture is governed by the maximum energy stored, as expressed in the following Equation 5.^60^

(Equation 5) $$U_{e}^{\max}=\frac{\sigma^{2}}{2E}=\frac{G^{2}K_{1G}^{2}}{2\pi a_{G}E}$$

**Figure 8:**
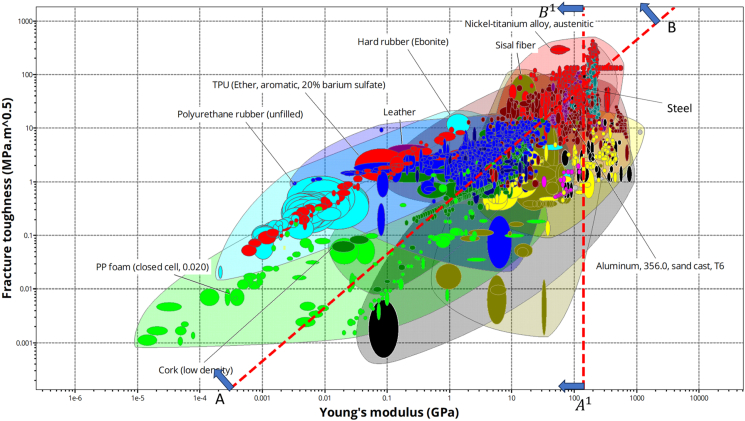
Ashby chart on fracture toughness vs. Young’s modulus Ashby chart showing the correlation between fracture toughness and Young’s modulus used for screening high-toughness materials for ballistic and dynamic impact applications. Figure adapted from Ref.^60^

Where “E” is the elastic modulus and $\left(\frac{K_{1G}^{2}}{E}\right)$ ≈ *J*_*G*_ is the critical toughness. The maximum energy absorption capability of a material improves with a higher value of critical toughness. For armor design, materials with high toughness and elasticity are preferred. The monolithic steel plate’s energy absorption capability was evaluated, and the guidelines “AB” and “A^1^B^1^” were plotted, considering the fracture toughness and elastic modulus of steel. The position above the guideline highlighted the better material candidates for armor design as demonstrated in Figure 8.

It can be concluded that, engineering composites and ceramics are the class of materials considered to be a suitable and it was conceptually screened. Areal density of monolithic steel materials for armor design was reduced exponentially, by considering composites and ceramic materials for design. According to the NIJ armor standard, for hard armor’s back face signature should not deform by more than 44 mm.^19^ Additionally, armor provides protection from danger, and after an impact event, shattered sections emerge. According to requirements, armor also had to be able to survive multiple bullets strikes. The design of modern armor relies on a variety of material phases; in all cases, hybrid composites are highly advised, as determined by conceptual design.

### Multi-criteria decision-making method and problem defining criteria’s considered for materials selection in fiber-reinforced polymer composites for ballistic application

FRPCs often experience damage mechanisms such as fiber breakage, delamination, and buckling failure under high-impact loading, limiting their applicability. To address these challenges, extensive research focuses on enhancing their dynamic performance by optimizing material composition, geometric parameters (fiber orientation and stacking sequence), loading conditions, and environmental factors.^61^

Ballistic material properties, including intrinsic parameters such as fabric characteristics (fabric type, weave pattern, and density) and yarn mechanical properties, play a significant role. Additionally, extrinsic factors such as projectile type and design, target configurations, ricochet impact, and ballistic testing methodologies are critical considerations. These factors significantly influence the behavior of fabric-based composites under ballistic impact scenarios.^62^ To identify the optimal composite panel, Gray Relation Analysis was employed, by considering four essential criteria: maximum directional deformation, energy absorption, maximum stress, and weight. In compliance with the NIJ standard, these parameters are guided for evaluation. The ideal panel demonstrated maximum energy absorption, minimal weight, and the least deformation.^63^ Key bullet attributes, including size, shape, velocity, and impact angle, must also be considered during armor design, as these characteristics impact deformation types, failure mechanisms, perforation depth, and perforation area in various composite materials. For Ultra-High-Molecular-Weight Polyethylene (UHMWPE) laminates with thicknesses of 10, 20, and 30 mm, experimental results revealed that the average energy absorption coefficient increases with laminate thickness. Testing of 20–30 mm thick laminates demonstrated progressive stages of mushroom-shaped bullet deformation, while a 10 mm laminate showed obvious penetration with considerable bullet damage.^64^

In the automotive industry, natural fibers such as kenaf and jute are increasingly preferred due to their advantageous mechanical properties, environmental benefits, and ability to meet vehicle performance requirements. However, the moisture absorption tendencies of natural fibers can compromise their durability. Consequently, for automotive applications, fibers with low moisture absorption properties are preferred. Moreover, if the cost of natural fibers remains competitive, they present a sustainable alternative to synthetic fibers, given their biodegradability and reduced environmental footprint. On the other hand, synthetic fibers, such as glass and carbon, are widely used due to their exceptional strength-to-weight ratios and durability. These materials provide superior mechanical properties, including high strength and stiffness, which are critical for load-bearing automotive components. Synthetic fibers also offer excellent resistance to environmental factors such as heat, chemicals, and moisture, although their higher cost can be a limiting factor for cost-sensitive applications.^65^ For fiber-reinforced composites, the choice of polymer matrix is equally critical, as it affects the overall performance of the composite. The matrix must ensure compatibility with the fiber for efficient load transfer, provide thermal stability, maintain flexibility for impact absorption, and be cost-effective and readily available for large-scale production. Mechanical properties such as strength, stiffness, and impact resistance are essential to ensure the reliability of automotive components under prolonged operational loads, as seen in applications such as engine mounts.^12^^,^^66^ Simultaneously, corrosion resistance, weight efficiency, and reduced environmental impact are crucial for performance optimization, particularly in high-impact applications. While Kevlar, glass, and carbon fiber composites deliver exceptional strength, their higher costs often restrict usage. In contrast, jute or kenaf-reinforced polymers offer cost-effective and environmentally friendly alternatives. Furthermore, polymer matrices such as high-density polyethylene (HDPE) or thermoplastic elastomers provide the required flexibility and impact resistance for automotive applications.^67^

Researchers have traditionally employed statistical tools to evaluate materials for design, based on selected performance criteria relevant to specific applications.^68^^,^^69^ Multiple attributes serve as design criteria for decision-making in material selection. Hybrid MCDM techniques such as integrated F-AHP and F-TOPSIS enhance decision-making accuracy, proving effective in selecting optimal compressors in petrochemical plants for long-term reliability. The closeness coefficient was used to gauge efficiency, showing that these methods offer a realistic model for equipment selection.^70^ Table 1 demonstrates the different MCDM approaches considered for different engineering application by defining PDC’s.

**Table 1:** MCDM methods are followed in different literature

MCDM Method	Application	Problem Defining Criteria	Reference
TRIZ (Theory of Inventive Problem Solving)	Conceptual design of automotive engine rubber mounting composites	Design goals, material properties, vibration absorption, durability, and environmental impact	Azammi et al.^65^
Analytic Network Process (ANP)	Selection of the best design concept for polymer composite engine rubber mounts	Performance, weight, cost, and material properties	
Preference Selection Index (PSI)	Optimal design of needle punched nonwoven fiber reinforced epoxy composites	Mechanical properties, erosive wear rate, density, and void content	Singh et al.^71^
TOPSIS (Technique for Order of Preference by Similarity to Ideal Solution)	Sustainable material selection problem	Environmental impact, cost, mechanical properties, and sustainability criteria	Mousavi-Nasab, and Sotoudeh-Anvari^67^
TOPSIS (Technique for Order of Preference by Similarity to Ideal Solution)	Sustainable material selection problem	Environmental impact, cost, mechanical properties, and sustainability criteria	Mousavi-Nasab, and Sotoudeh-Anvari^67^
Analytic Hierarchy Process (AHP)	Material selection for various applications, including automotive components	Performance attributes, cost, availability, and environmental impact	Mansor et al.^72^
TOPSIS (Technique for Order Preference by Similarity to Ideal Solution)	Crashworthiness analysis of bio-inspired thin-walled tubes based on Morpho wings microstructures	Energy absorption, peak crush force, crush force efficiency	Nikkhah et al.^73^
Analytical Hierarchy Process (AHP)	Selection of natural fibers for hybrid Kevlar®/natural fiber reinforced composites for personal body armor	Fiber orientation, cellulose content, cost, availability, density, tensile strength, and Young’s modulus	Naveen et al.^74^
TOPSIS (Technique for Order of Preference by Similarity to Ideal Solution)	Selection of the best laminate design for low-velocity impact performance	Areal density, deformation, absorbed energy, peak loading, and impact energy levels	Rezasefat et al.^75^
Grey-TOPSIS	Optimization of drilling parameters for CFRP machining	Surface roughness, uncut fiber, delamination, feed rate, twist angle, and lubrication type	Tran et al.^76^
Entropy Weighting	Determining the weight of decision-making criteria for drilling parameters	Importance of each criterion in the context of drilling performance	

Environmentally friendly lightweight materials are widely adopted in automotive applications, enhancing performance, reducing fuel consumption, and supporting sustainability. The entropy-weighted MAIRCA method, offering a simpler mathematical approach and strong evaluative capability, identified ultra-high strength steel blended composites as the most suitable material for such applications.^63^ Through AHP-MOORA, Patnaik et al.^77^ assessed the best composite for wear-resistant and structural use, with AHP providing criteria weights and MOORA for ranking. The composition of 30 wt. % 400 GSM viscose fabric mat, 15 wt. % BFS, and 55 wt. % epoxy was found optimal. Similarly, Bahraminasab et al.^78^ applied the VIKOR method to select optimal materials for total knee replacements, emphasizing the need to maintain aseptic properties. Their findings indicated that porous NiTi shape memory alloy outperformed the traditionally used Ti-6Al-7Nb as the best material choice. In another application, Swain et al.^79^ utilized the fuzzy-TOPSIS method to optimize NiTi plasma spray coating on mild steel, with a primary gas flow of 45 lpm and plasma arc current of 550A found to be optimal. According to Al-Oqla et al.^80^ AHP analysis ranked flax as the most suitable natural fiber for automotive uses, while coir, date palm, hemp, and sisal also demonstrated relevance. From the detailed literature on the MCDM approach for materials selection in ballistic applications hybrid MCDM approach was not largely considered for material selection in ballistic impact studies.

**Figure 9:**
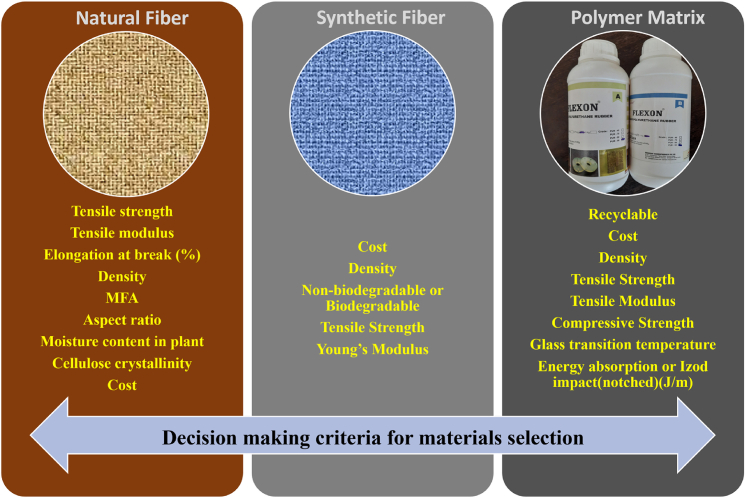
Problem defining criteria for ranking material for design

**Figure 10:**
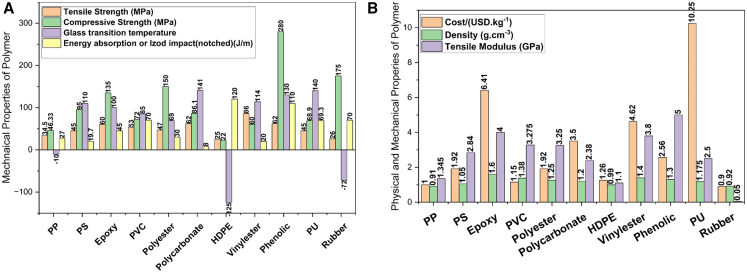
Physical, economical, and mechanical properties considered for polymer matrix selection through MCDM (A) Physical properties, (B) economical parameters, and (C) mechanical performance criteria considered for polymer matrix selection using Multi-Criteria Decision-Making (MCDM) techniques for fiber-reinforced composites. Data compiled from Refs.^81^^,^^82^^,^^83^

**Figure 11:**
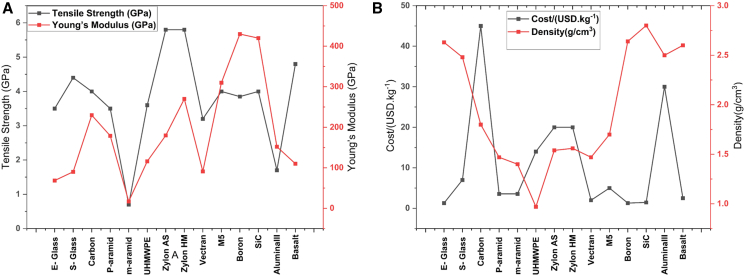
Physical, economical, and mechanical properties considered for Synthetic fiber (A) Physical properties, (B) economical factors, and (C) mechanical characteristics analysed for selecting suitable synthetic fibers in ballistic and high strain rate applications. Adapted from Refs.^84^^,^^85^

**Figure 12:**
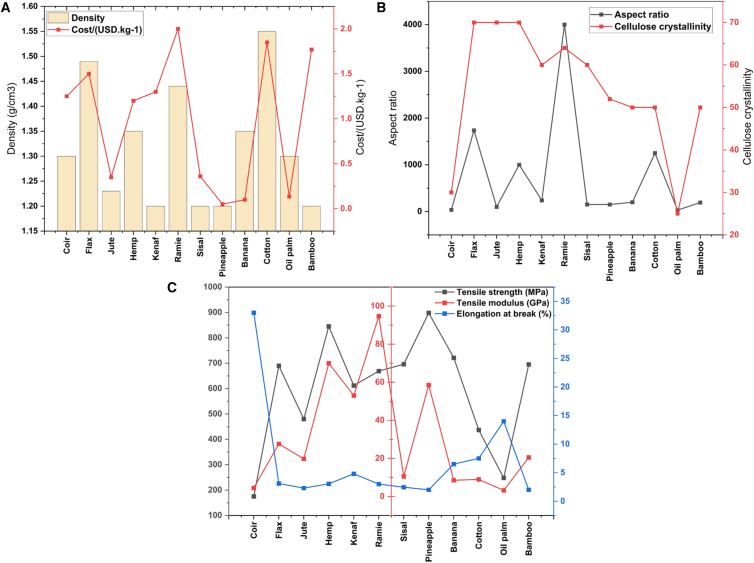
Physical, economical, and mechanical properties considered for natural fiber selection (A) Physical property criteria (density, moisture uptake, thermal stability), (B) economical criteria (cost, availability, processing effort), and (C) mechanical criteria (tensile strength, modulus, fracture toughness) used to screen natural fibers for composite applications. Data and criteria compiled from Refs.^3^^,^^80^^,^^86^

When designing FRPCs laminates for armor applications, selecting appropriate fibers and polymer matrices presents significant challenges. The criteria for polymer matrix selection typically include cost, density, recyclability, tensile strength, tensile modulus, compressive strength, glass transition temperature, and impact strength, tailored for specific applications. Similarly, criteria such as tensile modulus, tensile strength, elongation at break, density, microfibril angle, aspect ratio, moisture absorption, cellulose crystallinity, and cost are used for selecting synthetic fibers. For abundant natural fibers, biodegradability, cost, density, tensile strength, and modulus are considered critical design criteria. Figure 9, Figures 10, Figure 11 and 12 illustrate the PDCs for polymer matrices, synthetic fibers, and natural fibers, emphasizing their potential for ballistic impact applications through the application of MCDM techniques, as seen in other domains.

### Sustainability

The construction and automotive industries are increasingly adopting sustainable practices to comply with regulations and meet consumer demand for eco-friendly products. This shift includes using green materials, enhancing energy efficiency, and minimizing greenhouse gas emissions, aligning with sustainable development goals in ballistic armor and combat vehicles to balance economic growth with environmental responsibility.^43^^,^^71^^,^^87^

A key strategy for sustainability in lightweight structural materials is bio-composites, derived from renewable resources such as flax, hemp, jute, and bio-based polymers such as natural rubber, polylactic acid, and bio-epoxy. These materials offer an alternative to traditional composites with benefits in renewability, biodegradability, and reduced carbon footprint, particularly in military vehicles where weight reduction improves fuel efficiency and performance.^2^^,^^53^^,^^87^ Additionally, fiber composites require less energy to manufacture, making them cost-effective and environmentally friendly.

Advancements in FRPC manufacturing, such as compression molding, injection molding, and additive manufacturing (FDM, FFF, SLS) are improving efficiency and sustainability.^88^ Innovations in biodegradable matrices and hybridizing synthetic fibers such as carbon, basalt, and Kevlar with natural hemp, jute, and flax enhance mechanical properties, energy absorption, and recyclability.^89^ Life cycle assessments and waste reduction through recycling further support a circular economy.^90^ Challenges such as moisture absorption, fiber variability, and matrix compatibility remain. Addressing these through hybrid fiber composites, chemical treatments, and interfacial modifications can enhance performance and market adoption.^5^ Recent advances demonstrate that engineering the fiber/matrix interphase can significantly enhance composite performance. De-sizing treatments of CF in CF/PEEK composites raised IFSS by ∼25%, leading to marked gains in ILSS (up from 62.5 to 71.3 MPa), flexural (up to ∼805 MPa), tensile strength (∼37%), strain-to-failure (∼190%), and energy absorption (∼230%).^91^ Nanomaterial interfaces e.g., with nanocellulose or CNT coatings also improved stiffness and strength by 15–33%, while polyaniline-GNP hybrid interlayers achieved a striking 79% improvement in mode-I fracture toughness.^92^ These findings mirror the benefits of strong interfacial adhesion in delaying delamination and enhancing ballistic energy dissipation.

Policies and collaborations across defense, aerospace, and automotive sectors encourage sustainable innovation through funding, tax incentives, and recycling mandates. International partnerships, such as those within NATO, facilitate standardization, while public-private collaborations support research and commercialization.^93^ Future efforts focus on global standard harmonization, bio-material subsidies, and supply chain strengthening to accelerate high-performance composite adoption.^4^

Beyond combat vehicles, fiber composites are valuable in high strain rate applications such as aerospace, marine, and construction sectors. In aerospace, they contribute to fuel efficiency, while in marine applications, their corrosion resistance extends vessel longevity.^53^ Despite challenges in recycling natural fiber-reinforced composites, advances in mechanical, chemical, and biological recycling technologies are improving material recovery, reducing waste, and supporting closed-loop systems in defense and automotive industries.^43^^,^^87^ The integration of biodegradable polymers, such as polylactic acid, polyurethanes, polyethylene glycol, and chitosan, further enhances recyclability.^2^^,^^53^

**Figure 13:**
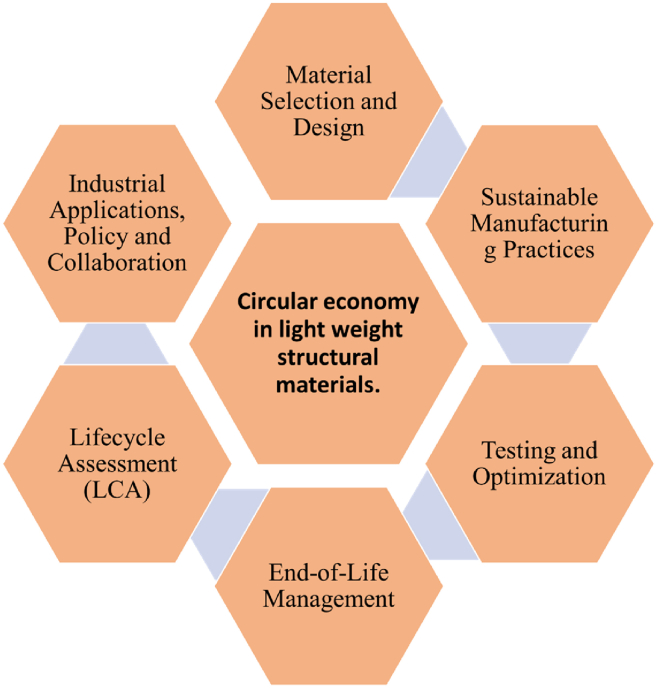
Circular economy in FRPCs for ballistic application

In the marine and construction sectors, fiber composites promote sustainability, while in ballistic protection, they present lightweight alternatives for armor.^2^ Even in textiles, natural fibers such as jute, flax, coir, ramie, kenaf, pineapple, and hemp contribute to sustainable FRPCs for body armor, reducing waste and improving material quality.^94^ Figure 13 illustrates the role of FRPCs in circular economy-driven ballistic applications. Replacing glass fiber with flax or hemp can reduce the Global Warming Potential (GWP) by up to 50%, lowering emissions from ∼10 to 15 kg CO_2_-eq/kg for glass/epoxy to 3–5 kg CO_2_-eq/kg for natural fiber composites, while cutting cumulative energy demand from ∼150 to 200 MJ/kg to 50–90 MJ/kg due to the lower processing energy of plant fibers. These materials also present lower water consumption and reduced toxic emissions. At end-of-life, thermoplastic-based natural FRPCs achieve >90% recyclability through mechanical processing with minimal property loss, whereas thermoset systems can still recover fibers via pyrolysis or chemical depolymerization, albeit with a 20–40% reduction in properties.^18^^,^^95^ Embedding these quantified metrics into the review not only substantiates the environmental advantages of bio-based and hybrid composites but also offers a performance sustainability decision framework that accounts for carbon footprint, embodied energy, and recyclability. However, large-scale adoption remains limited due to variability in material properties, durability concerns, and the need for further optimization under stringent ballistic standards.

## Design and geometry

This section discusses various stacking configurations in FRPCs considered for high-strain rate SHPB tests, as well as low and high-velocity impact studies.

**Figure 14:**
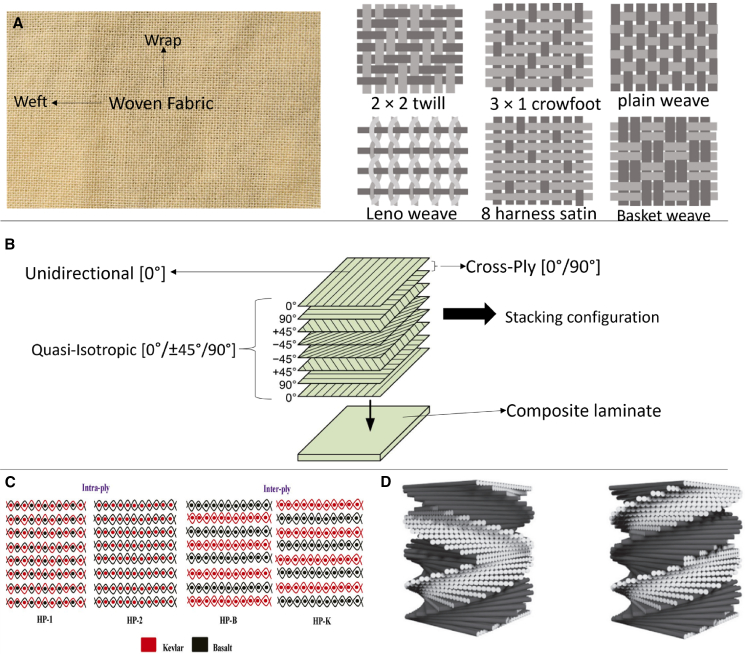
Different stacking configurations considered for LVI, SHPB, and HVI impact tests Representative stacking and weaving configurations (unidirectional, cross-ply, quasi-isotropic, helicoidal, and hybrid) investigated for low-velocity impact (LVI), Split Hopkinson Pressure Bar (SHPB), and high-velocity impact (HVI) testing of fiber-reinforced composites. Figure adapted from Refs.^27^^,^^96^^,^^97^

Cross-ply laminates consist of reinforcing fibers oriented at 0° and 90°, fibers arranged in warp and weft perpendicularly, and denoted as [0°/90°]. Quasi-isotropic laminates feature multiple layers at 0°, 90°, and ±45°, stacked symmetrically from the mid-plane to achieve uniform properties in different directions. The fiber orientation of helicoidal laminates, a kind of quasi-isotropic laminate with symmetric and non-symmetric stacking configuration, fiber axis rotates along the laminate’s thickness, potentially reducing delamination and enhancing mechanical performance.^2^^,^^8^ Figure 14 illustrates the various fabric stacking and weaving patterns considered for the high strain rate experiments, while Table A1 in Appendix A presents the effect of stacking configurations on SHPB performance on different loading conditions. The arrangement of fibers, the behavior of the matrix, and the associated failure modes significantly influence composite performance under high-strain rate conditions. These factors play a crucial role in optimizing composite materials for dynamic applications.

### Stacking configuration influence on the split Hopkinson pressure bar compression test

The stacking sequence significantly influences the compressive strength of composites. Unidirectional laminates generally exhibit higher compressive strength than cross-ply configurations, due to their fiber alignment in the loading direction. Under dynamic loading, both peak stress and modulus increase with strain rate, with dynamic modulus reaching 2.5 to 4.5 times the static modulus.^97^ This enhancement is attributed to the limited time for material deformation, resulting in brittle failure modes and higher peak stresses due to reduced energy dissipation.^97^^,^^98^ Qian et al.^99^ reported an increase in out-of-plane peak compressive stress from 456 MPa (quasi-static) to 782 MPa at increasing strain rate. They also demonstrate that cross-ply laminates [0°/90°] enable multidirectional load distribution, enhancing damage tolerance and energy absorption.

Ravikumar et al.^100^ studied E-glass/epoxy laminates, showing that variations in fiber orientation significantly impact compressive strength and failure modes. Satin weave patterns, due to tighter interlacing, reduce shear failure risks under dynamic loads.^97^^,^^101^ High strain rate tests on woven Kevlar composites highlight the role of fiber-matrix interfaces in enhancing strength and energy dissipation.^102^ In hybrid fiber composites, such as carbon/Kevlar laminates, outperform monolithic Kevlar in stiffness and toughness properties under dynamic compression test.^103^ Strategic stacking, such as satin weave carbon combined with plain weave E-glass, further improves compressive strength and structural resilience due to its synergistic fiber effect.^104^

Bio-inspired helicoidal configurations and advanced stacking designs, such as ±45° orientations, have shown improvements in interlaminar shear strength by mitigating brittle failure modes such as delamination.^29^ Gowda et al.^105^ observed that basalt/hemp/PU rubber composites with helicoidal stacking exhibit superior energy absorption and shear resistance under SHPB impact. They demonstrate that increased fiber content and ply-layer sequence also contribute to higher peak stresses, as the viscoelastic rubber matrix delays fiber failure under rapid loading.^99^^,^^106^^,^^107^ Stitched composites with Z-fiber reinforcement introduce through-thickness strength, improving shear and interlaminar failure resistance. SHPB testing shows that these 3D-reinforced composites outperform traditional 2D woven laminates under dynamic loads.^108^ Strain rate sensitivity, a defining trait of composites, shifts failure modes from ductile to brittle with increasing strain rates, progressing from shear banding to fiber breakage and delamination.^100^ Configurations such as ±45° or helicoidal laminate provide alternate load paths, boosting overall high strain rate performance by improving brittle crack mitigation.

External environmental conditions, especially temperature, further influence dynamic behavior. Carbon/epoxy laminates soften at elevated temperatures, transitioning to ductile failure. SHPB studies affirm that both strain rate and temperature critically impact performance.^109^ Across laminate such as AS4/PEEK, woven Kevlar, and hybrid satin-carbon/E-glass laminates, stacking sequence and fiber orientation remain key to optimizing dynamic compressive performances.^104^^,^^110^^,^^111^

**Figure 15:**
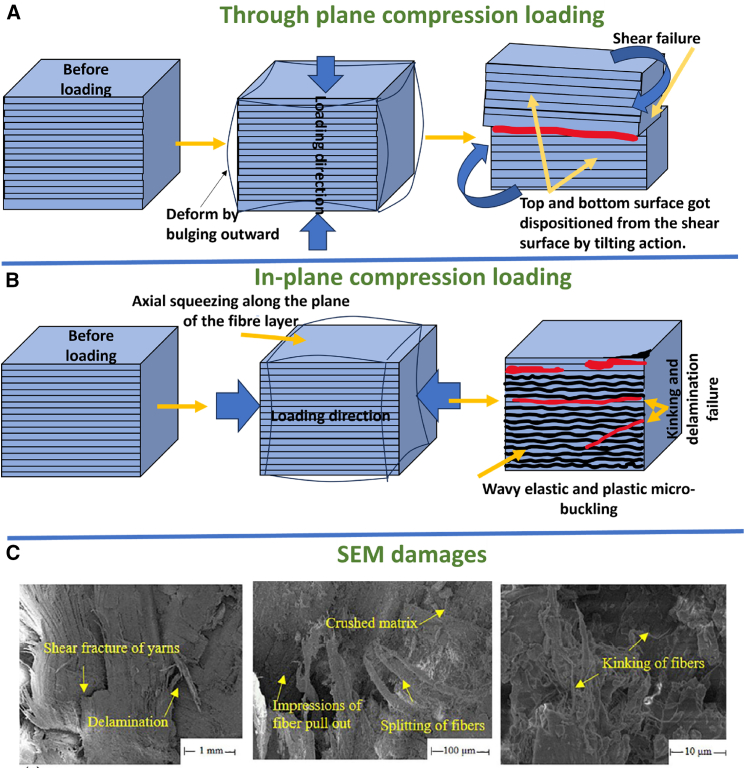
SHPB test setups and SEM fractography of impacted specimens (A) SHPB compression test setup and schematic of specimen orientation, (B) SHPB testing in tensile/off-axis modes (representative fixture and gauge locations), and (C) representative SEM fractography showing fiber breakage, matrix cracking and interfacial debonding after high-rate loading. Data and criteria compiled from Ref.^103^

**Figure 16:**
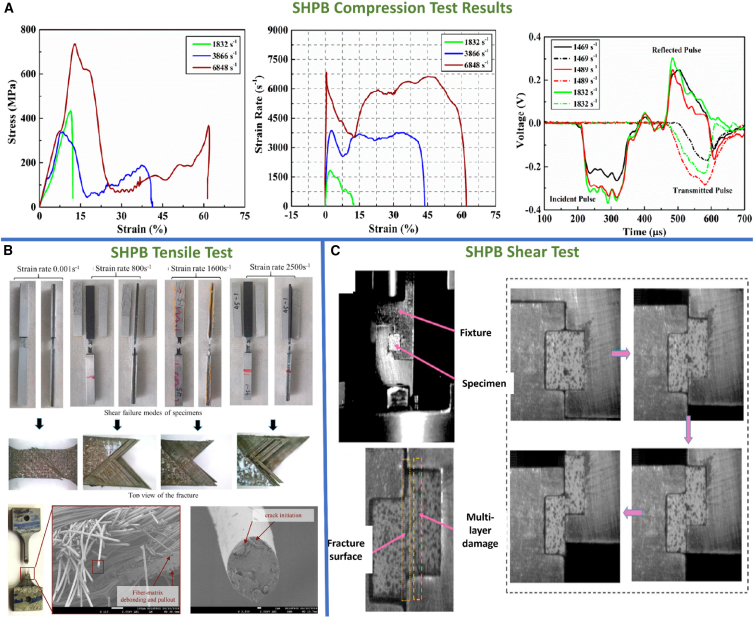
SHPB dynamic test examples and observed damage modes (A) Typical high-strain-rate stress–strain response from SHPB compression tests, (B) failure patterns observed in SHPB tensile specimens (necking, fiber pull-out), and (C) shear-mode specimens showing interlaminar sliding and shear-dominated failure. Data and criteria compiled from Refs.^117^^,^^118^^,^^119^

Axial bulging and in-plane squeezing effects during SHPB compression loading on fiber composites are illustrated in Figures 15A and 15B, showing failure modes such as kinking and shear delamination. The investigated samples experience kinking band failure effects and shear delamination as resulted.^112^^,^^113^^,^^114^^,^^115^^,^^116^ SEM fractography by Priyanka et al.^103^ revealed features including yarn shear fracture, matrix crushing, fiber splitting and pull-out, and kinking (Figure 15C). Figure 16A demonstrates rising peak stress and strain in AS4/PEEK laminates with increased strain rates, indicating the dynamic load withstanding capability of composites. During SHPB testing, strain gauge signals recorded by an oscilloscope (Figure 15A) capture incident, reflected, and transmitted pulses, used to compute stress-strain responses.^117^ Overall, Fiber composites demonstrate stacking sequence, loading direction, and material sensitivity on SHPB compression test performance at ambient temperature.

### Stacking configuration influence on the split Hopkinson pressure bar tension test

As like in compression, tensile strength and modulus of composite laminates are greatly influenced by their stacking sequence. Research demonstrates that SHPB mechanical properties, such as axial strength, often improve with increased strain rate.^118^^,^^120^

The influence of composite structure on tensile results obtained from the SHPB test is complex, encompassing factors such as material composition, fiber orientation, strain rate sensitivity, and failure mechanisms. Composites with fibers aligned in the loading direction (0°) exhibit higher tensile strength than those with fibers at angles such as ±45°, as load transfer efficiency varies with fiber alignment.^121^^,^^122^ Strain rate sensitivity is another critical aspect, with materials such as carbon fiber-reinforced plastics (CFRP) demonstrate tensile strength increases from 40% to 80% under dynamic loading compared to quasi-static conditions. This behavior depends on the composite’s structure, strain energy absorption, speed of testing, and stress distribution enabled by the fiber-matrix interaction.^123^^,^^124^

Failure mechanisms during SHPB testing transition from fiber pull-out and matrix cracking at lower strain rates to matrix cracking and fiber breakage at higher rates, as shown in Figure 16B. The structural configuration of composites, such as hybridization or fiber type, significantly influences these mechanisms.^123^^,^^125^^,^^126^ Recycled office paper (ROP) and old corrugated cardboard (OCC) fiber composites demonstrated varying strain rate sensitivities based on fiber composition, with different failure patterns such as fiber breakage and changes in fiber curvature being observed.^125^ Micromechanical models, which consider fiber and matrix contributions, validate these behaviors, with predictions aligning with experimental data. These models demonstrate the importance of fiber properties such as cellulose content, in influencing the tensile performance of natural fibers.^121^^,^^125^

FEA simulations further elucidate the stress distribution and failure mechanisms of different composite configurations under SHPB loading, providing a valuable tool for understanding and optimizing composite performance.^123^ Experimental studies on materials such as CFRP and hybrid natural fibers confirm that structural configuration, strain rate sensitivity, and failure mechanisms are interdependent, dictating the composite’s overall tensile behavior under dynamic loading conditions.^124^^,^^125^ These insights are crucial for designing high-performance composites for applications requiring resilience under impact and dynamic loads.

Unidirectional IM7/8551-7 graphite/epoxy composites, tensile strength was observed to decrease as fiber orientation shifted from the longitudinal (0°) to transverse (90°) direction, with the highest sensitivity to strain rate seen in the longitudinal direction due to improved load transfer efficiency when fibers align with the loading direction.^127^ Moreover, the matrix’s viscoelastic properties further enhance tensile response under dynamic loading, as it stiffens with increased strain rate, supporting the composite’s ability to resist deformation.^101^

### Stacking configuration influence on split Hopkinson pressure bar shear test

In adhesively bonded composite joints, shear strength was affected by hygrothermal aging and moisture absorption, leading to matrix plasticization, which initially increases ductility but can reduce shear strength under dynamic conditions.^128^ This plasticization influences the matrix stiffness and load transfer, affecting interfacial bonding and ultimately altering shear performance.

Furthermore, fiber architecture, including stitching and stacking arrangement, can significantly affect shear behavior, especially in woven composites. Stitched composites experience reduced micro-buckling and delamination, which enhances shear strength.^101^ Empirical models derived from high-speed tensile tests have also demonstrated that in-plane shear properties are more sensitive to strain rate variations than interlaminar shear properties, emphasizing the role of layup configuration.^119^ Research on carbon fiber reinforced aluminum laminate (CARALL) composites has highlighted that stacking configurations, such as woven layers, critically influence failure modes under dynamic loading. Although CARALL is not highly strain rate sensitive in terms of strength, it exhibits significant changes in failure mechanisms, emphasizing the role of stacking configuration on mechanical responses.^129^

Overall, studies show that shear strength in fiber composites generally increases with strain rate, with layup configurations such as ±45° for in-plane and 0° for interlaminar shear proving critical in high-strain scenarios. These configurations not only impact shear strength and modulus but also affect the nature of failure, making stacking design essential in applications subject to dynamic loads.^119^^,^^130^

**Figure 17:**
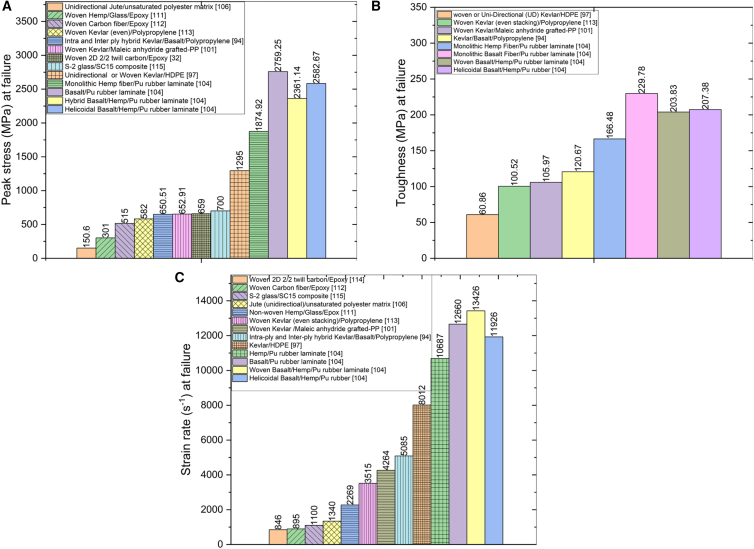
Comparative high strain-rate performance across laminate configurations (A) Peak stress comparison for different stacking/architecture (UD, cross-ply, quasi-isotropic, helicoidal, hybrid), (B) toughness (energy absorbed to failure) comparison, and (C) strain-rate sensitivity trends across configurations. Data summarized from reviewed studies.

Figure 17 illustrates the enhancement in peak stress, toughness, and strain rate-dependent properties obtained from Split Hopkinson Pressure Bar (SHPB) testing of various laminate configurations reported in the literature. Among the studied configurations, helicoidal laminates reinforced with hemp and basalt fibers embedded in a polyurethane (PU) rubber matrix exhibit significantly superior dynamic performance. These helicoidal structures outperform traditional laminates such as monolithic fiber laminates, unidirectional (UD), woven, twill, even-numbered layer stacking, as well as hybrid configurations with intra-ply and inter-ply architectures.

The superior SHPB response observed in helicoidal laminates can be attributed to the progressive rotation of the fiber orientation layer-by-layer, which facilitates efficient stress redistribution and energy dissipation under high strain rate loading. Moreover, the integration of a compliant matrix, such as polyurethane rubber, contributes to enhanced toughness and resistance to catastrophic failure by delaying crack initiation and propagation. This viscoelastic nature of the rubbery matrix plays a crucial role in absorbing impact energy and mitigating brittle failure modes typically associated with high strain rate events.

Consequently, for dynamic loading applications such as impact, blast, or ballistic environments, the use of elastomeric matrices combined with natural and synthetic hybrid fibers arranged in a helicoidal architecture presents a promising strategy to improve structural resilience and energy absorption capability. This design approach not only enhances mechanical performance but also offers improved safety and durability in high-strain-rate applications.

### Low velocity impact

**Figure 18:**
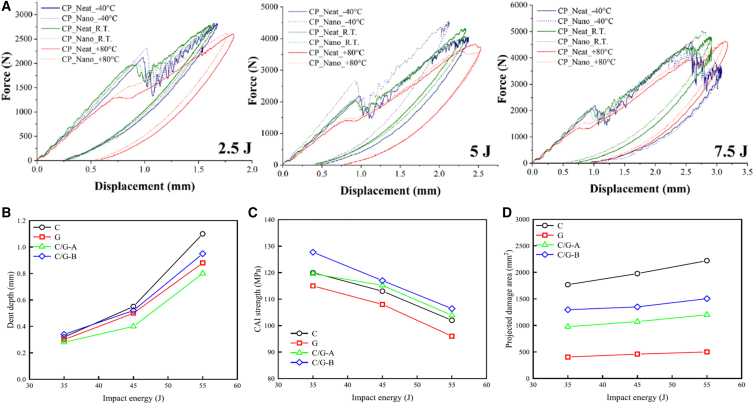
Impact force–displacement and residual strength metrics (A) Force vs. displacement curves for laminates at varying impact energies, (B) peak force and dent depth trends, (C) Compression-After-Impact (CAI) residual strength variation with impact energy, and (D) photographic/CT view of damage morphology at representative energies. Data and criteria compiled from Ref.^131^

The influence of stacking sequence, fiber hybridization, impact energy, and architectural configuration on the low-velocity impact (LVI) performance and energy absorption characteristics of composite materials has been extensively studied at ambient temperature. Table A2 in Appendix A summarizes the effects of stacking configurations on LVI properties for various composite systems. The combined effect of laminate design and testing parameters, such as temperature, on energy absorption under LVI conditions is illustrated via the force-displacement curve shown in Figure 18A. Fiber composites demonstrate lower peak force with higher residual displacement, due to the softening of the polymer matrix at higher temperatures. At ambient and lower temperatures, a clear primary delamination force drop was observed due to the brittle nature composite. The hormonal variation in trend due to delamination crack propagation across the lamina. Additionally, the depth of penetration and the extent of damage area tend to increase with higher initial impact energy, as demonstrated in Figures 18B–18D. Furthermore, an increase in impact energy and variation in the laminate configuration often led to a decrease in compression-after-impact (CAI) residual strength.^131^

Jambhulkar and Sahu^132^ demonstrated that a hybrid laminate composed of Kevlar and glass fabrics in a K/K/G/G/K stacking increased energy absorption by 16.68% while reducing overall cost by 32% compared to monolithic Kevlar fiber laminates. Hazzard et al.^133^ highlighted the significant influence of fiber orientation on deformation mechanisms. Cross-ply laminates (0°/90°) exhibited larger back-face deflections due to in-plane shear deformation, forming a four-sided pyramid-shaped impact zone, while quasi-isotropic architectures reduced central deflection by 43% through extensive panel buckling and shear concentration. Helicoidal laminates displayed a combination of wrinkling and buckling, where decreasing ply angle mismatch led to increased wrinkling, suggesting better deformation accommodation but potentially lower ballistic failure resistance compared to quasi-isotropic counterparts.

Lopes et al.^134^ proposed dispersed stacking sequences to improve damage tolerance, and Saleh et al.^50^ investigated non-crimp fabric (NCF), 2D plain weave, and 3D woven architectures. Among these, 3D woven composites exhibited superior damage tolerance and residual strength due to the presence of z-binding yarns, which suppressed delamination and promoted progressive failure. Jiang et al.^135^ explored bio-inspired helicoidal laminates featuring non-linear rotation angle-based stacking sequences. Their findings indicated that Helicoidal-Recursive and Helicoidal-Exponential configurations employing larger rotation angles offered improved impact resistance and higher threshold loads for damage initiation compared to quasi-isotropic laminates.^29^

Caminero et al.^136^ studied the stacking sequence’s effect on Charpy impact and flexural damage behavior. Unidirectional laminates absorbed more energy than multidirectional ones, which displayed complex failure patterns due to fiber orientation coupling. Ricciardi et al.^137^ found that flax/basalt hybrid epoxy laminates benefited from stacking sequence optimization, showing enhanced flexural and interlaminar shear strength as well as better impact resistance and damage tolerance. Asaee and Taheri^138^ studied 3D fiber metal laminates (3DFMLs) comprising magnesium alloy plate and 3D fiberglass fabrics, concluding that stacking sequence strongly affected stiffness, strength, and energy absorption. The 3DFML-4-2/2 configuration exhibited superior peak contact force and energy absorption over 3DFML-10 at equal weight and cost.

Wang et al.^139^ examined hybrid carbon/flax composites with sandwich-like stacking (carbon/flax/flax/flax/carbon). The stronger carbon fiber skin enhanced impact performance and adhesion, while the stacking sequence influenced damping behavior and energy consumption. Wu et al.^140^ demonstrated that non-uniform braided architectures offered superior impact resistance compared to uniform designs, thanks to improved stress distribution. Hadj-Djilani et al.^141^ observed that cross-ply and quasi-isotropic flax/epoxy laminates outperformed angle-ply structures in terms of flexural and displacement properties due to fiber orientation along the loading axis. While void defects influenced damage mechanisms, their presence did not significantly affect overall impact resistance.

Sergi et al.^131^ revealed that quasi-isotropic CFRP laminates with multi-walled carbon nanotube (MWCNT) reinforcement had enhanced damage tolerance and residual flexural strength. MWCNTs delayed delamination crack propagation by reinforcing the matrix against opening. In hybrid flax/basalt/aluminum metal-joint laminates, Umar bin Ashraf et al.^142^ found that asymmetric basalt/flax stacking led to superior impact resistance and structural integrity, supporting hybridization benefits. Lyu et al.^143^ further affirmed that hybrid stacking sequences influence damage mechanisms and CAI strength, showing hybrid configurations outperforming monolithic ones due to synergistic fiber interactions. Lopes et al. and Others^134^^,^^144^^,^^145^ demonstrated that dispersed stacking sequences improved damage tolerance and reduced delamination, offering a more reliable design for composite structures. El Moumen et al.^146^ showed that carbon nanotube (CNT) reinforcement enhanced energy absorption and rigidity in CFRPs, with optimal CNT content reducing the damage zone.

Chen et al.^147^ investigated interlayer hybrid composites made from carbon, glass, and basalt fibers. Their results showed that configurations with carbon fiber cores offered the highest impact resistance due to reduced flexibility. Similarly, Kazemi et al.^148^ analyzed thermoplastic and thermosetting hybrid composites, noting that UHMWPE outer layers in thermoplastic hybrids minimized structural loss and absorbed more impact energy. These laminates displayed extended plasticity and lower absorbed energy (by 48%) than their thermoset counterparts. Bandaru et al.^149^ found that alternating basalt and Kevlar layers in thermoplastic composites improved peak force and energy absorption during impact, mitigating the weaker compressive strength of Kevlar through hybrid synergy. Studies by Sun et al.^150^ and Lebaupin et al.^151^ reiterated the importance of stacking sequence, where quasi-isotropic laminates offered better damage tolerance than cross-ply designs, albeit with more distributed damage. A higher aramid fiber content raised the critical penetration velocity, while aramid fibers effectively absorbed impact energy and reduced back-face failures. Masud and Mubashar,^152^ emphasized the role of symmetric stacking and fiber hybridization in enhancing impact behavior in carbon/flax bio-hybrid laminates. Tiyek and Kaya^153^ reported that nonwoven interlayers reduced deformation and improved impact resistance by halting crack propagation and boosting ductility. Charca et al.^154^ found that complex weaves such as basket patterns enhanced energy absorption through architectural reinforcement effects. Pandian et al.^155^ concluded that specific configurations, such as 3/2/3-layer jute/glass/jute stacking, improved energy absorption while limiting damage. Jute fiber composites showed better energy absorption but larger damage zones than glass fiber ones. Finally, Nikkhah et al.^73^ studied bio-inspired multi-layered tubes modeled after Morpho butterfly wings, demonstrating that such structures offered improved crashworthiness and energy absorption compared to traditional architectures.

Collectively, these studies demonstrate that optimizing fiber stacking, hybridization, reinforcement techniques, and incorporating innovative materials such as nanoparticles or bio-inspired designs are crucial in enhancing the dynamic performance of composite materials, particularly in high-performance applications in automotive, aerospace, and defense sectors.

### High velocity impact

The ballistic performance of hybrid thermoplastic/thermosetting composite armors is highly influenced by stacking configuration and fiber architecture. Bandaru et al.^156^ found that symmetric stacking enhanced ballistic resistance, with composites in such configurations withstanding impact velocities above 395 m/s, while non-symmetric stacking failed at 365–395 m/s, increasing the ballistic limit by 26.27%. In a follow-up study, Bandaru et al.^157^ demonstrated that 3D-angle interlock fabrics, incorporating z-yarns for improved interlaminar strength, absorbed 36% more energy at the ballistic limit than 2D woven fabrics due to reduced delamination. Signetti et al.^158^ similarly observed that symmetrical Kevlar/Innegra/carbon composites yielded superior ballistic performance by promoting uniform stress distribution, in contrast to asymmetric sequences that led to stress concentrations and reduced toughness.

**Figure 19:**
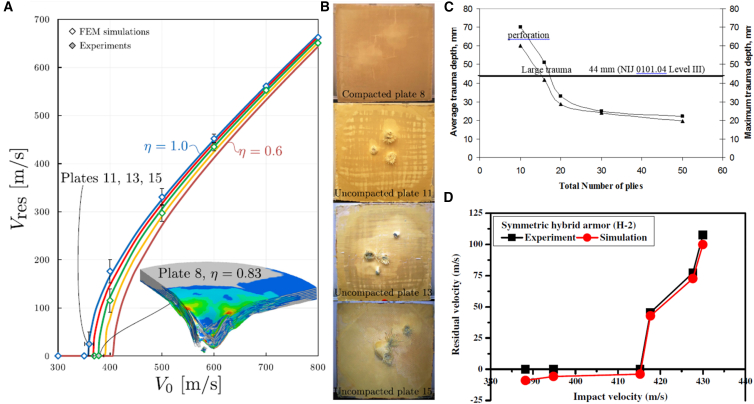
Residual velocity, trauma depth and FE validation of HVI tests (A) Residual velocity vs. initial projectile velocity curves, (B) images of failed/damaged panels after HVI, (C) average trauma (BFS) depth vs. number of plies/stacking sequence, and (D) comparison of experimental and FE predicted residual velocities (model validation). Data and criteria compiled from Refs.^156^^,^^159^^,^^160^

Laminates exhibit varying ballistic limit thresholds and damage pattern, corresponding to the relationship between the bullet’s initial velocity and its residual velocity as demonstrated in Figures 19A and 19B. Hybrid material systems also benefit from tailored stacking and synergistic effects. Palta et al.,^14^ showed that Kevlar/steel composites exploited different failure mechanisms to enhance energy dissipation, raising the ballistic limit by 26% over monolithic materials. Ramadhan et al.^161^ reported that placing aluminum plates at the rear of Kevlar-29/epoxy laminates in hard armor optimizes energy absorption through mechanisms such as deformation, delamination, and shear failure. Peinado et al.^17^ further emphasized the role of stacking in UHMWPE composites, achieving a 31% performance boost by combining different fiber layers with varying ultimate strains while maintaining similar areal densities.

Traditional soft armors typically use the symmetric stacking of orthotropic fabrics. However, recent research has explored angular stacking, where layers are oriented at varied angles (e.g., [0°/30°/60°] for three layers and [0°/22.5°/45°/67.5°] for four layers), enabling multidirectional stress wave propagation and enhancing energy absorption by 19%–58%. The incorporation of shear thickening fluids (STF) has further improved impact performance by engaging more fibers under the high shear rates effect of bullet.^162^ Multi-layered armor systems combining different materials in optimized sequences can minimize damage from penetration and spalling. Acoustic impedance matching between layers is also key in mitigating transmitted stress waves.^163^ High-specific modulus fiber composites, such as those using Kevlar (KF), glass (GF), and carbon fiber (CF), show marked dependence on stacking order. Optimal arrangements, such as placing CF at the front and KF at the rear, enhance energy dissipation and reduce ballistic limit velocity.^164^ Similarly, strategic stacking influences impact strength and failure mechanisms such as fiber breakage and matrix cracking.^159^ In Kevlar/UHMWPE hybrids, both ply number and arrangement are critical. A 16-ply configuration was identified as optimal, with fewer plies correlating to increased trauma depth and reduced protection, as shown in Figure 19C.^160^ The effects of stacking configuration on high-velocity impact (HVI) behavior across various composite systems are summarized in Table 2.

**Table 2:** Stacking configuration influence on HVI test results

Laminates types	Structure types	Stacking	Effect on performance	Reference
Kevlar® Reinforced Thermoplastic	3D Orthogonal	[0°/±45°/90°]	Improved energy absorption and damage tolerance; higher ballistic limit compared to 2D structures.	Bandaru et al. and Bandaru et al.^157^^,^^165^
Carbon Fiber Reinforced Polymer (CFRP)	2D Non-Crimp Fabric	[0°/90°]	Lower impact performance; prone to delamination under ballistic impact.	Chocron et al.^166^
Carbon Fiber Reinforced Polymer (CFRP)	3D Woven Composite	3D woven fabric	Enhanced damage tolerance; reduced damage area compared to 2D composites.	
Hybrid Fiber-Reinforced Epoxy Composites	Non-hybrid and hybrid configurations (Kevlar® (K), carbon (C), glass (G))	K/K/K, C/C/C, G/G/G, K/G/K, K/C/K, G/C/K, C/G/K, K/C/G	Stacking sequence affects flexural strength; Kevlar®^/^epoxy showed the highest energy absorption; damage mechanisms include delamination and fiber breakage.	Stephen et al.^167^
Basalt Fibers/Epoxy Composites with Graphene nano particles (GNPs)	Basalt fiber reinforced epoxy with graphene nanoplatelets	Various weight percentages of GNPs (0, 0.1, 0.2, 0.3, 0.4, 0.5)	Maximum improvement in impact limit velocity and energy absorption at 0.3 wt. % GNPs; enhanced interfacial characteristics between fibers and matrix.	Kazemi-Khasragh et al.^168^
Aluminum/Jute Fibers-Epoxy Composites with Nano-clay	Fiber metal laminates (FMLs) with aluminum and jute fibers	0°/90°/0°/90°/0° configuration with varying nano-clay percentages (0, 1, 3, 5 wt. %)	Improved impact properties with 3 wt. % nano-clay; reduced delamination length and enhanced adhesion between layers; optimal performance at 3 wt. % nano-clay.	Ebrahimnezhad-Khaljiri et al.^169^
Carbon/Natural Fiber Hybrid Composites	Interply and Intraply hybrids	Various configurations	Improved toughness and energy absorption; hybridization modifies failure modes, enhancing damage tolerance.	Santulli et al.^170^
Kevlar®^/^Rubber Composite	Multi-layered with a rubber matrix	Two-layer and four-layer configurations	Rubber matrix enhances energy absorption and flexibility; better ballistic performance compared to thermoset matrix composites.	Khodadadi et al.^171^
Kevlar®/Epoxy Composite	Multi-layered with a thermoset matrix	Two-layer and four-layer configurations	Thermoset matrix restricts fabric deformation, leading to lower energy absorption and ballistic performance compared to rubber matrix composites.	
Hemp/Flax Hybrid Bio-composite	Nonwoven mats and unidirectional (UD) prepregs	Various stacking sequences (e.g., UD, mat)	Stacking sequence significantly affects impact performance; multiple layers of UD prepregs enhance impact resistance.	Baysal et al.^172^
Hybrid Long Carbon/Glass Fiber Composite	Long fiber thermoplastic composite	Various fiber lengths (5, 10, 20 mm)	Increased fiber length improves tensile strength and impact resistance; hybridization enhances overall performance compared to monolithic glass fiber composites.	Shayan Asenjan et al.^173^

The study of composite laminates and hybrid materials for ballistic applications has garnered increasing interest, with numerous investigations examining the effects of stacking sequence, fiber architecture, and material composition under high-velocity impact. Jambhulkar and Sahu^132^ showed that hybrid laminates comprising woven kenaf and aramid fibers reinforced with polyvinyl butyral (PVB) film outperformed pure aramid composites, particularly when kenaf and aramid layers were strategically stacked. This configuration not only enhanced energy dissipation but also offered a cost-effective and eco-friendly solution. Sabadin et al.^174^ evaluated composite armor with silicon carbide (SiC) ceramic front layers and UHMWPE fiber backings, revealing that optimal layer arrangement significantly boosted ballistic resistance by combining the hardness of ceramics with the flexibility of UHMWPE for superior energy absorption. Similarly, Stephen et al.^167^ found that Kevlar/carbon/glass fiber hybrid epoxy composites exhibited stacking-sequence-dependent performance, with rear-positioned Kevlar plies offering the best energy dissipation. Wei et al.^175^ compared stacked cross-plied composite fabrics (SCCF) to cross-plied laminated panels (CLP), finding SCCF superior in energy absorption, while CLP retained greater structural integrity. Ma et al.^176^ further highlighted that three-dimensional angle-interlock woven fabrics (3DAWF) and their reinforced composites (3DAWC) demonstrated distinct ballistic behaviors based on impact velocity, with 3DAWC performing better at high velocities due to improved energy transfer through the resin matrix.

**Figure 20:**
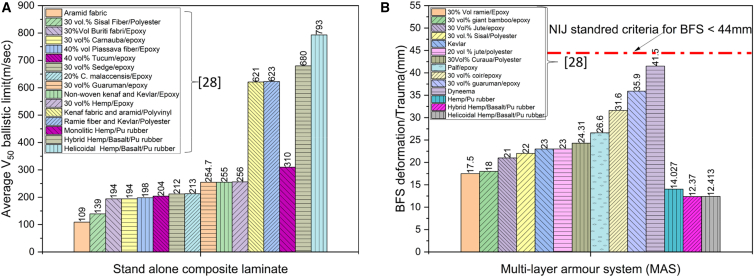
Comparative ballistic performance (V50 and BFS) across laminates (A) V50 ballistic limit for a range of composite laminates and hybrid systems, and (B) corresponding Back-Face Signature (BFS) deformation measurements used to assess trauma in hard armor tests.

Figure 20 presents a comparative assessment of the ballistic performance of various composite laminates in terms of the V_50_ ballistic limit and Back Face Signature (BFS) trauma, evaluated for both soft and hard armor systems. The design criteria of 44 mm BFS deformation recommended for hard armor as indicated in the figure. Comparatively with Kevlar laminates, hybrid natural and synthetic fiber composites demonstrate an eight times improvement in V_50_ ballistic limit was observed. The results indicate that composites with a reinforcement content of up to 30–40 wt. % exhibit superior performance when integrated within compliant matrices such as rubber and elastomers, particularly when compared to their counterparts fabricated with conventional brittle matrices, including epoxy, polyvinyl, polyethylene, and polyester.

Notably, helicoidal structured laminates reinforced with hybrid fibers and embedded in soft matrices demonstrate significantly enhanced ballistic resistance. These bio-inspired architectures achieved higher V_50_ values, indicating greater projectile resistance while simultaneously reducing BFS deformation, which is critical in minimizing trauma in personal protection systems. The gradual variation in fiber orientation through the laminate thickness enables a controlled dispersion of stress waves and efficient absorption of kinetic energy, thereby mitigating localized damage and delamination that typically compromise the integrity of traditional composites under high-velocity impact.

Collectively, these findings highlight the critical role of tailored stacking sequences, fiber hybridization, and matrix compliance in optimizing the energy dissipation mechanisms of composite armors. The synergy between structural design and material selection proves instrumental in improving both the ballistic limit and wearer safety. Such advances underscore the potential of helicoidal hybrid composites in next-generation defense applications, offering an effective balance between lightweight construction, impact resistance, and structural resilience.

## Simulation and modeling

In this section discussed on finite element analysis (FEA) is discussed, it has become an indispensable tool in simulating the ballistic and high strain rate behavior of composite materials, enabling accurate predictions of damage mechanisms, energy absorption, and structural integrity under extreme conditions.

**Figure 21:**
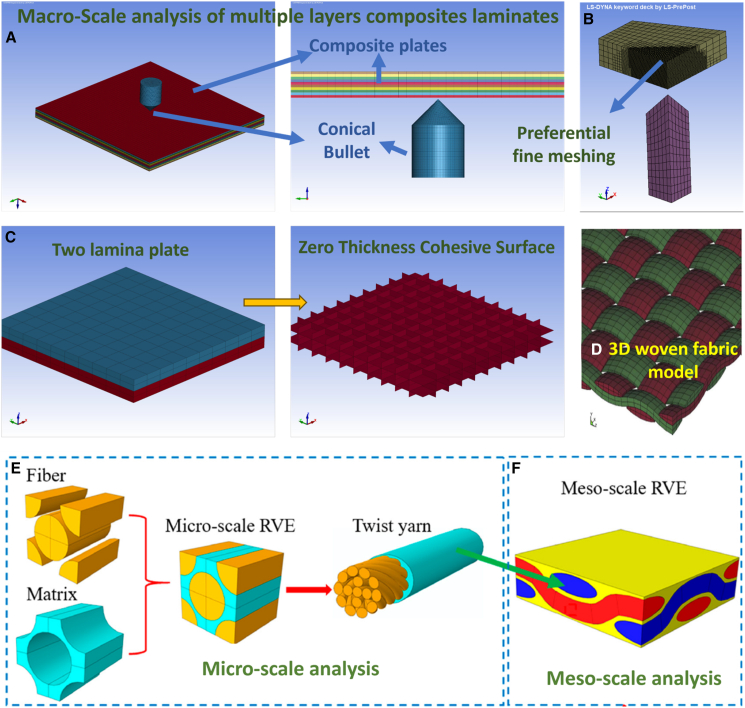
Multi-scale finite element modelling approaches for impact (A) Macro-scale FE model example, (B) fine meshing strategy for local damage capture, (C) zero-thickness cohesive-surface representation for delamination, (D) 3D woven fabric modeling approach, (E) micro-scale representative volume element (RVE), and (F) meso-scale modeling for yarn-level response. Adapted from cited modelling studies. Data and criteria compiled from Refs.^49^^,^^177^

Tools such as ABAQUS, ANSYS AUTODYN, and LS-DYNA are widely utilized for modeling composite materials, using advanced material models such as orthotropic constitutive equations, continuum damage models (Hashin damage, composite damage, and plastic kinematics), and cohesive zone models to replicate the anisotropic nature of composites. These models help capture delamination, matrix cracking, and fiber breakage, which are typical damage phenomena during ballistic impacts. The Johnson-Holmquist (JH2) model has been suggested for progressive damage simulation of ceramic plates in Kevlar-epoxy composites,^157^^,^^161^ and validation against experimental data, such as in Peinado et al.,^17^ confirms strong correlations in energy absorption prediction for UHMWPE panels impacted by steel projectiles. Developments in material design and computational modeling continue to improve laminate performance, reduce fabrication costs, and time, and enhance the understanding of composite behavior in protective applications. Figure 19D illustrates the accuracy of FEA predictions at various high-velocity impact (HVI) conditions, while Figure 21 displays different scale models, including fine and coarse mesh, zero-thickness cohesive surface modeling, and macro-, meso-, and micro-scale representative volume elements. Table A3 in Appendix A presents details of different FEA models, testing setups, software packages, and evaluated properties.

Several researchers have applied FEA tools in designing advanced protective composite armor. Arora et al.^162^ used ABAQUS and LS-DYNA to simulate the ballistic response of neat and shear-thickening fluid (STF)-impregnated fabrics, revealing insights into stacking sequence effects on stress wave propagation. Similarly, Signetti et al.^159^ employed LS-DYNA and a continuum damage model to simulate woven fabric composites, highlighting the necessity of capturing anisotropic behavior for accurate predictions. Pai et al.^163^ emphasized the importance of advanced material models in understanding the failure mechanisms and energy absorption in multi-layered armor systems. Jambhulkar and Sahu^132^ used ANSYS with orthotropic models to simulate interactions between Kevlar and glass fabrics, successfully validating their predictions against experiments. Across these studies,^132^^,^^159^^,^^162^^,^^163^ validation using experimental data was consistently emphasized to ensure simulation reliability.

Sabadin et al.^174^ used ANSYS AUTODYN for simulating dynamic projectile impacts on armor systems using both mesh and meshless methods. Their model predicted BFS deformation, ceramic fragmentation, bullet deformation, and fracture with high accuracy, validated by experimental ballistics tests. Stephen et al.^167^ showed that placing Kevlar layers at the rear of hybrid composites improved impact resistance, a result aligned with physical tests. Wei et al.^175^ applied LS-DYNA to aramid fiber composites, confirming the significance of fiber orientation and stacking sequence in energy absorption. Jiang et al.^135^ built a 3D ABAQUS/Explicit model to simulate drop-weight tests on bio-inspired helicoidal laminates, using progressive damage models to capture both intra- and inter-ply failure. Asaee and Taheri^138^ applied ABAQUS/Explicit with VUMAT subroutines to predict the response of 3D fiber metal laminates (FMLs) under impact, while Lyu et al.^143^ developed a computational model incorporating strain and Tsai-Wu criteria to estimate damage and residual compressive strength in hybrid laminates. Other significant contributions using ABAQUS include studies by Hadj-Djilani et al.,^141^ Sergi et al.,^131^ Lopes et al.,^134^ and El Moumen et al.,^146^ who utilized failure models such as Hashin, Chang-Chang, and continuum damage mechanics (CDM) to simulate impact damage in flax/epoxy and CFRP composites.

Further expanding the capabilities of FEA, Chen et al.^147^ combined CDM with cohesive zone models (CZM) in ABAQUS to predict both intraply damage and delamination in hybrid laminates, achieving excellent correlation with experiments. Sujon et al.^178^ and Kazemi et al.^148^ modeled hybrid thermoplastic composites and incorporated VUMAT subroutines with the Chang-Chang damage model, accurately simulating low-velocity impacts and validating the model against experiments.^149^ Tiyek and Kaya^153^ assessed the response of nonwoven reinforced composites using a CDM-CZM approach, stressing the importance of fiber-matrix interactions. Sun et al.^150^ used CDM to simulate damage accumulation in laminated composites under repeated impacts, while Pandian et al.^155^ explored damage mechanisms such as matrix cracking and delamination in glass-jute composites using ABAQUS. Nikkhah et al.^73^ applied LS-DYNA to model the crush behavior of bio-inspired tubular structures, achieving strong agreement with physical tests in energy absorption prediction. Finally, Ma et al.^176^ focused on the ballistic behavior of 3D woven fiber composites (3DAWF and 3DAWC), highlighting the matrix’s role in dissipating impact energy. Collectively, these studies underscore the crucial role of FEA in capturing the complex dynamic response of composite materials, offering valuable insights for optimizing structural configurations in aerospace, automotive, and defense applications.

### Composite damage model

Matrix failure under tensile and compressive loading is assessed using the Tsai-Wu criteria, whereas the Chang-Chang criteria define the failure mechanisms for tensile and compressive fiber modes.^179^^,^^180^^,^^181^ Accordingly, the fiber tensile mode is analyzed for failure types.

(Equation 6) $$\sigma_{aa}\;>0\;\Rightarrow \;e_{f}^{2}={\left(\frac{\sigma_{\text{aa}}}{X_{t}}\right)}^{2}+\beta {\left(\frac{\sigma_{\text{ab}}}{S_{c}}\right)}^{2}-1$$

(Equation 7) $$e_{f}^{2}\geq 0\;\Rightarrow \text{Failed}$$

(Equation 8) $$e_{f}^{2}<0\;\Rightarrow \text{Elastic}$$

(Equation 9) $$E_{a}=E_{b}=E_{c}=G_{ab}=\nu_{ba}=\nu_{ab}=0$$

For the compressive fiber mode:

(Equation 10) $$\sigma_{aa}<\;0\;\Rightarrow \;{\left(\frac{\sigma_{\text{aa}}}{X_{c}}\right)}^{2}-1$$

(Equation 11) $$e_{c}^{2}\geq 0\;\Rightarrow \text{Failed}$$

(Equation 12) $$e_{c}^{2}<0\;\Rightarrow \text{Elastic}$$

(Equation 13) $$E_{a}=\nu_{ba}=\nu_{ab}=0$$

For the tensile and compressive matrix mode:

(Equation 14) $$e_{md}^{2}=\frac{{\sigma_{\text{bb}}}^{2}}{Y_{c\;}Y_{t}}+{\left(\frac{\sigma_{\text{ab}}}{S_{c}}\right)}^{2}+\left(\frac{\left(Y_{c\;}-Y_{t}\right)\sigma_{\text{bb}}}{Y_{c\;}Y_{t}}\right)-1$$

(Equation 15) $$e_{md}^{2}\geq 0\;\Rightarrow \text{Failed}$$

(Equation 16) $$e_{md}^{2}<0\;\Rightarrow \text{Elastic}$$

The post-damage behavior and material degradation follow the definition of the *SOFT* parameters. When one of the failure modes is initiated from the Chang-Chang failure criteria, the strength reduction factors, or damage factors, get involved with the material strength properties when the matrix starts to crack. Fiber tensile strength softening factor *(FBRT)* and fiber compressive strength softening factors *(YCFAC)* are described by the following equations:

(Equation 17) $$X_{t^{1}}=X_{t}\times \text{FBRT}$$

(Equation 18) $$X_{c^{1}}=Y_{C}\times YCFAC$$

When the stresses in each ply approach the failure strain thresholds, the elements may also be deleted. The maximal tensorial shear strain and the tensile failure strain in the fiber direction are known as *DFAILS* and *DFAILT* in the case of unidirectional tape. Whereas *DFAILC* and *DFAILM* are the compressive failure strain in fiber direction and maximum strain for tensile or compressive matrix straining, respectively. In this investigation, the equations that result from dividing the strength by the corresponding modulus were used to determine the strain values as reported by,^28^ as summarized below:

(Equation 19) $$DFAILT=\frac{X_{t}}{E_{a}}$$

(Equation 20) $$\text{DFAIL}C=\frac{X_{c}}{E_{a}}$$

(Equation 21) $$DFAILM=\frac{Y_{t}Y_{c}}{E_{b}}$$

(Equation 22) $$DFAILT=\frac{X_{t}}{E_{a}}$$

(Equation 23) $$DFAILS=\frac{S_{c}}{G_{ab}}$$

After the current row of components is removed, the crash front reduction factor *(SOFT)* in the material definition lowers the strength of the parts in the subsequent row.^179^ Since this is a non-physical cost-effective interpretation of the material’s damage zone, the SOFT parameter spans from 0 to 1, with 0 representing no strength drop in the model prior to failure.

### Ceramic plate material model

The Johnson-Holmquist-2 (JH-2) model provides a framework for understanding how brittle materials such as ceramics respond under dynamic and high-pressure conditions. By normalizing stresses and pressures against the Hugoniot Elastic Limit (HEL), it offers a means to study material behavior during impacts.

(Equation 24) $$\sigma^{*}=\sigma_{i}^{*}-D\left(\sigma_{i}^{*}-\sigma_{f}^{*}\right)$$

In the JH-2 model, stresses are normalized with respect to the Hugoniot Elastic Limit (HEL), σ_*HEL*_. The intact stress $\sigma_{i}^{*}$ and fractured stress $\sigma_{f}^{*}$ are described using material-specific parameters A, B, C, N, and M, while normalized pressure *P*^∗^ and tensile pressure *T*^∗^ are defined relative to HEL (*P*_*HEL*_). The evolution of material damage is captured by the damage variable D.

(Equation 25) $$\sigma_{i}^{*}=A{\left(P^{*}+T^{*}\right)}^{N}\;\left(1+Cln\left({\dot{\varepsilon }}^{*}\right)\right)$$

(Equation 26) $$\sigma_{f}^{*}=B{\left(P^{*}\right)}^{M}(1+Cln\left({\dot{\varepsilon }}^{*}\right)$$

(Equation 27) $$\varepsilon_{f}^{p}=D_{1}{\left(P^{*}+T^{*}\right)}^{D_{2}}$$

The failure strain $\varepsilon_{f}^{p}$, a critical parameter in the model, is described by constants *D*_1_ and *D*_2_. The hydrostatic pressure-density relationship is expressed through *μ* = $\frac{\rho }{\rho_{0}}$ – 1, where *ρ*_0_ is the reference density. Constants K_1_, K_2_, and K_3_ are used to describe the material’s compressibility, while an additional term ΔP accounts for dilation during failure.

(Equation 28) $$P=K_{1}\mu +K_{2}\mu^{2}+K_{3}\mu^{3}+\Delta P\;\left(\mu \geq 0\right)$$

(Equation 29) $$P=K_{1}\mu \left(\mu \leq 0\right)$$

(Equation 30) $${\Delta P}_{t+\Delta t}=-K_{1}\mu_{t+\Delta t\;}+\sqrt{{\left(K_{1}\;\mu_{t+\Delta t\;}+{\Delta P}_{t}\right)}^{2}+2\beta K_{1}\Delta U}$$

This model, implemented in Johnson_Holmquist_Ceramic, has been validated for ceramics such as Al_2_O_3_ and SiC using parameters provided by Ahmed et al.^182^ and Holmquist et al.^183^ The model also accounts for elastic energy dissipation (*β*) and its transformation into potential energy (ΔU).

### BFS plate material model

The MODIFIED_JOHNSON_COOK (MJC) model refines the original Johnson-Cook material model to provide improved accuracy in simulating material responses under severe conditions, such as high plastic deformation, rapid strain rates, and elevated temperatures.^28^^,^^147^ The flow stress (σ) in the original model is a function of strain (*ε*), strain rate ($\dot{\varepsilon }$), and temperature (T), with material parameters A, B, C, n, and m controlling the relationships.

(Equation 31) $$\sigma =\left(A+B\varepsilon^{n}\right)\left[1+C\;\ln\left(\frac{\dot{\varepsilon }}{{\dot{\varepsilon }}_{0}}\right)\right]\left[1-{\left(\frac{T-T_{room}}{T_{melt}-T_{room}}\right)}^{m}\right]$$

In dynamic loading scenarios, the fracture behavior of materials is modeled using an enhanced Johnson-Cook fracture model, which introduces the damage variable (D) to track the progression toward failure. This damage variable is defined as:

(Equation 32) $$D=\sum \frac{\Delta \varepsilon^{p}}{\varepsilon_{f}}$$

Here, Δ*ε*^*p*^ is the increment of effective plastic strain, and *ε*_*f*_ is the fracture strain. The fracture strain is a function of stress triaxiality (η), strain rate ($\dot{\varepsilon })$, and temperature (T), with constants D_1_, D_2_, D_3_, D_4_, and D_5_ specific to each material.

(Equation 33) $$\varepsilon =(D_{1}+D_{2}(\exp\left(D_{3}\eta \right)\left[1+D_{4}\;\ln\left(\frac{\dot{\varepsilon }}{{\dot{\varepsilon }}_{0}}\right)\right]\left[1-D_{5}\left(\frac{T-T_{room}}{T_{melt}-T_{room}}\right)\right]$$

Stress triaxiality (η), defined as the ratio of mean stress to equivalent stress, plays a critical role in the fracture process. Additional key variables include strain rate ($\dot{\varepsilon }$), reference strain rate (${\dot{\varepsilon }}_{0})$, temperature (T), room temperature (T_room_), and melting temperature (T_melt_).

## Manufacturing process

Fiber-reinforced polymer composites (FRPCs) are fabricated using a variety of procedures that have a substantial impact on environmental impact, scalability, cost, and performance. In this section, discussed on fabrication technique followed in the literature.

**Figure 22:**
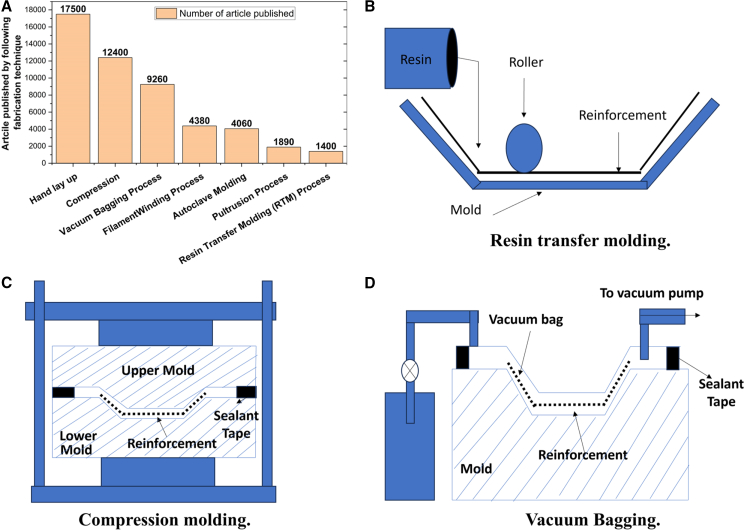
Publications & fabrication methods followed for FRPCs (A) Number of articles published by fabrication method (publication count, ScienceDirect), (B) common Resin Transfer Molding (RTM) examples and outputs, (C) compression molding sample descriptions, and (D) vacuum bagging examples used in laboratory production.

In fiber composites production, methods such as prepreg layup, resin transfer molding (RTM), and filament winding are commonly used.^184^ Prepreg layup involves pre-impregnated fibers partially cured and laid in a mold before full curing in an autoclave, offering high strength-to-weight ratios and excellent fatigue resistance, but with high costs due to costly raw materials and energy-intensive processes.^48^ In the RTM method, which injects resin under pressure into a mold containing dry fibers, ensures better fiber wet-out and reduced void content, making it suitable for medium to high-volume production with lower material waste compared to hand layup.^185^ RTM, compression, and the vacuum bagging method are illustrated schematically in Figures 22B and 22D. Filament winding, often used for cylindrical shapes, offers scalable production with low material waste but higher production costs.^83^ However, CFRP production incurs high costs between $230 and $310 per kilogram, mainly due to the expensive materials and the energy-intensive nature of the processes, impacting scalability and resulting in material waste during cutting and curing.^48^

For natural FRPCs production, compression molding and injection molding are common techniques. Compression molding offers faster production cycles and is suited for complex shapes, providing better scalability for medium to high-volume production with moderate material waste. Hand layup, a flexible method with a low initial investment, is suitable for low-volume production but labour-intensive, generating moderate material waste from excess resin and trimming.^83^^,^^186^ Natural FRPCs provide eco-friendly benefits due to their biodegradability, reduced energy requirements, and abundant raw materials, resulting in lower production costs compared to CFRPs.^187^ RTM, by controlling fiber wet-out and reducing void content, is more efficient for larger production runs, minimizing waste. Prepreg layup, although expensive due to the use of prepregs and autoclave processing, delivers high-quality composites with minimal waste and excellent mechanical properties.^186^ Injection molding, while highly scalable, offers a good surface finish but comes with higher initial equipment costs. In synthetic fiber reinforced composites, methods such as filament winding and braiding are common. Filament winding, ideal for cylindrical shapes, offers low material waste and moderate production costs, while braiding is suitable for medium to high-volume production but generates moderate waste.^83^

Hand layup and compression molding, though cost-effective, are less efficient for high-performance applications, while advanced methods such as RTM and prepreg layup offer superior mechanical properties at higher costs.^83^^,^^186^ Automated manufacturing techniques, including Automated Tape Layup (ATL) and Automated Fiber Placement (AFP), enhance precision and are suitable for large-scale production, reducing material waste through precise control over material placement.^188^ The choice of manufacturing method depends on balancing performance, cost, scalability, and sustainability, with hand layup and compression molding being suitable for low to medium volume production at lower costs but potentially limited mechanical performance. When compared to other methods, Figure 22A showed that the number of articles published (Science Direct) in these manufacturing techniques is substantial, making them the top priority for laboratory-scale research. In contrast, advanced methods such as RTM, prepreg layup, and automated techniques excel in terms of mechanical properties and scalability but come with higher costs and complexity.^185^^,^^188^ Future advancements in manufacturing technologies will be crucial for optimizing FRP composites for diverse applications and improving the sustainability of production processes.^189^

## Testing and validation

**Figure 23:**
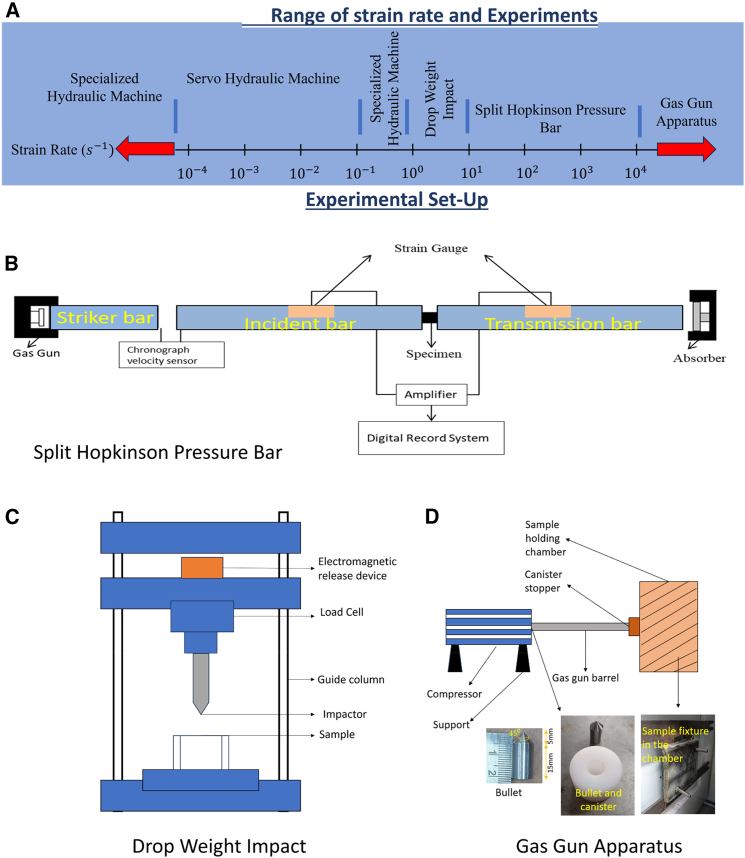
Experimental testing range and apparatus for dynamic loading (A) Range of strain rates and corresponding experimental techniques (LVI, SHPB, gas-gun/HVI, blast), (B) split-Hopkinson pressure bar (SHPB) setup for high strain-rate tests, (C) drop-weight (low-velocity impact) apparatus, and (D) gas-gun high-velocity impact (HVI) rig (compressor, barrel, canister, sample holder).

The different experimental techniques for a variety of strain rate tests under ambient temperature, as shown in Figure 23A, are covered in this section. An abundance of research is being done on servo hydraulic machines, drop weight impact, SHPB, and gas gun apparatus at strain rates (s^−1^) scale of 10^−4^-10^−1^, 1–10, 10-10^4^ and greater than 10^4^ on various materials.^109^

### SHPB tests

The mechanical behavior and damage mechanisms of composite materials under dynamic loading conditions have been extensively studied, with a focus on testing, validation, and predictive modeling. For sea shell-mimetic composites, Dutta et al.^190^ highlighted the importance of understanding dynamic self-stiffening behavior under high strain rates. Using a Split Hopkinson Pressure Bar (SHPB) apparatus as demonstrated in Figure 23B, the study validated an analytical model based on the load transfer between ceramic blocks and the polymer matrix through shear deformation. Finite element (FE) simulations employing the shear-lag theory confirmed the hierarchical structure’s role in damage resistance, with ceramic layers serving as load-bearing components and polymer layers facilitating load redistribution.

Similarly, Nunes et al.^191^ investigated adhesive joints under dynamic loading, with SHPB custom specimens developed to derive traction-separation laws (TSLs) and fracture energy in mode I and mode II conditions. These findings were validated against traditional methods such as Double Cantilever Beam (DCB) and End Notch Flexure (ENF) tests, showcasing comparable results. Cohesive zone modeling (CZM) in FE simulations revealed a transition from elastic to plastic behavior in ductile adhesives, emphasizing the need to accurately model stress distribution during damage initiation and propagation. High strain rate tensile loading of E-glass/epoxy composites by Kumar and Naik^120^ employed variable rate power law formulations to predict tensile strength, validated by SHPB tests. The study showed a strong correlation between analytical predictions and experimental data, emphasizing the reliability of the proposed model. In parallel, Tsai and Sun developed a visco-plasticity model for S2/8552 glass/epoxy composites, confirming its applicability across strain rates through stress-strain SHPB comparisons.^192^

Additionally, the compressive behavior of AS4/3501-6 carbon/epoxy composites was investigated by Bing and Sun^193^ using off-axis specimens with titanium coatings to reduce friction. The study developed a nonlinear, rate-dependent constitutive model, validated against high strain rate tests. The compressive strength increased significantly from 649.7 MPa at low strain rates (10^−5^/s) to 934.9 MPa at higher rates (250/s), with model predictions aligning closely with experimental results. These findings collectively emphasize the interplay between material architecture, testing methodologies, and FE modeling in understanding damage mechanisms under dynamic loading. Experimental validations using SHPB tests, coupled with robust analytical and FE models, provide critical insights into optimizing the mechanical behavior of composites for high-performance applications, particularly in protective materials, aerospace, and automotive sectors.^31^^,^^120^^,^^190^^,^^191^^,^^192^^,^^193^^,^^194^^,^^195^

### Low velocity impact

Low-velocity impact (LVI) testing is a crucial method for evaluating the energy absorption capabilities and damage resistance of composite laminates, especially in aerospace applications.^47^ Key performance metrics, such as maximum energy absorption and the extent of damage, including delamination, are extensively studied to optimize composite designs for impact-critical applications through a drop weight impact apparatus as shown in Figure 23C. Low velocity impact performance for different stacking laminates at different energy impacts was given in the Table 3.

**Table 3:** Low velocity impact performance of different configured laminates

Laminate Type	Maximum Energy Absorption	Area of Damage and FE model	Key Findings	Reference
Helicoidal Composites	Higher than traditional structured laminates	Increased delamination area but lower degree of perforation, it improves the residual strength of composites. Area of damage predicted by the composite damage material model.	Enhanced energy absorption and damage tolerance compared to quasi-isotropic laminates.	Ginzburg et al.^27^
T700GC/M21 Composite	Varies (up to 25 J)	Larger damage area for higher the initial impact energy predicted by Hashin and Puck criteria.	FE results are good correlation with experimental; effective in predicting damage mechanisms.	Li et al.^196^
Hybrid 3D Woven Composite	30 J–79 J at 94 J and 162 J initial impact	Reduced delamination compared to 2D laminates. Area of damage predicted by cohesive elements (delamination) and truss elements (z- yarns).	Enhanced energy absorption and damage tolerance due to fiber 3D architecture.	Muñoz et al.^51^
Composite Plates	Varies (6 J, 10 J, 13 J)	Accurate prediction of delamination area by cohesive elements.	Robust FE model validated against experimental data; effective for LVI simulations	Lamanna, and Caputo^197^
Various Composite Laminates	Varies (21 J–39 J)	Projected delamination area was predicted accurately by the scale effect	Validated analytical scaling method for damage area prediction	Bogenfeld et al.^198^
Quasi-Isotropic Laminates	15 J–35 J	Cohesive zone modeling approach correlation with experimental delamination damage	Developed an efficient FE model; accurately predicted impact and compression strength	Joglekar et al.^199^
Cross-Ply Composite Laminates	7.35 J, 11.03 J, 14.70 J	Larger delamination areas predicted through the cohesive zone model	Modified progressive damage model; included through-thickness stress for better accuracy of results.	–
AS4/8552 CFRP Laminates	Varies (up to 70 J)	Mapped delamination accurately predicted by the cohesive zone model	Efficient numerical approach for CAI strength; predictions within 5% of experimental results	Baluch et al.^200^
Carbon Fiber-Reinforced Composites	Varies (5 J–20 J)	Delamination and matrix cracks identified and validated through cohesive zone modeling	NDT techniques used for damage reconstruction; validated against numerical simulations	Katunin et al.^36^
Carbon Fiber Laminates	Varies (up to 70 J)	Mapped delamination accurately predicted by cohesive zone modeling	Reviewed semi-empirical models; validated against experimental results.	Antonucci et al.^201^
Thin Composite Laminates	4 J, 6 J, 10 J, 15 J	Increased delamination with higher impact energy predicted by cohesive zone modeling	Developed a 3D damage model; good correlation with experimental results.	Tuo et al.^202^
Hybrid Laminates (C/G/B)	50 J	Damage area increased with impact energy predicted by cohesive zone modeling	Hybrid laminates showed superior impact resistance; they validated the FE model against experimental data.	Chen et al.^147^
Carbon Fiber Laminates	∼50 J	Mapped delamination accurately predicted by cohesive zone modeling	Validated simulations against experimental results; effective in predicting damage mechanisms	English et al.^203^
Composite Plates	6 J, 10 J, 13 J	Intra-lamina and inter-laminate damage captured through cohesive zone modeling	Numerical simulations validated against experimental tests; good correlation in damage predictions	Caputo et al.^204^
FRPC armor	N/A	Rib fractures and pelvic injuries predicted	Validated a full human body model against lateral impact tests; good agreement in force and injury predictions	Vavalle et al.^205^

Maximum energy absorption is a vital parameter for assessing a laminate’s ability to withstand impact. Baluch et al.^200^ demonstrated a numerical model capable of predicting the Compression After Impact (CAI) strength of carbon fiber-reinforced polymer (CFRP) laminates, achieving predictions within 5% of experimental results, thus highlighting its reliability. Figure 18C demonstrates significant decreases in CAI residual strength with increases in initial impact energies, due to an increase in the area of damage and dent penetration. Antonucci et al.^201^ and Tuo et al.^202^ reviewed semi-empirical and numerical approaches, revealing that hybrid laminates incorporating carbon fibers as the core exhibited superior energy absorption compared to non-hybrid counterparts. Similarly, Katunin et al.^36^ showed that energy absorption is significantly influenced by laminate configuration and impact energy levels.

Comparative studies have also explored material-specific energy absorption capabilities. Schoeppner and Abrate^206^ observed that IM7/5260 laminates exhibited higher delamination threshold loads (DTLs) and superior energy absorption compared to AS4/3501-6 and AS4/APC-2 laminates under similar impact conditions. Bio-inspired helicoidal laminates further demonstrated enhanced energy absorption, minimizing through-thickness failures compared to traditional quasi-isotropic and cross-ply laminates.^27^ These findings align with Faggiani and Falzon,^207^ who developed FE models that closely matched experimental observations, capturing both energy absorption and impact force with high accuracy.

Delamination area, a critical indicator of structural integrity post-impact, increases with impact energy and is strongly influenced by stacking sequence.^35^ Baluch et al.^200^ effectively integrated delamination mapping into their FE models, simplifying damage prediction by focusing on critical zones. Katunin et al.^36^ reconstructed delamination profiles using ultrasonic scanning and CT imaging, while English et al.^203^ employed similar techniques to verify the accuracy of simulations in capturing matrix cracks and fiber breaks. The role of fiber architecture in damage resistance has also been highlighted. Muñoz et al.^51^ demonstrated that hybrid 3D woven composites showed reduced delamination compared to traditional 2D laminates. Helicoidal laminates, despite higher delamination in LVI tests, maintained superior residual strength in CAI tests.^27^

Robust FE modeling has proven critical in correlating experimental and simulated results. Lamanna and Caputo^197^ developed FE models that accurately captured interlaminar and intralaminar damage, showing strong alignment with experimental data across various impact energies. Similarly, Tuo et al.^202^ introduced a 3D damage model incorporating interlaminar and intralaminar mechanisms, achieving excellent correlation with experimental results. Antonucci et al.^201^ emphasized the necessity of tailored approaches in laminate design to ensure structural reliability. English et al.^203^ and Caputo et al.^204^ demonstrated that refined FE approaches could predict force-time curves, damage growth, and structural responses with high precision. Baluch et al.^200^ and Katunin et al.^36^ further emphasized the importance of integrating experimental and simulation findings for enhanced accuracy in damage prediction.

Collectively, these studies emphasize the importance of optimizing stacking sequences, fiber architectures, and material combinations to enhance energy absorption and damage resistance in composite laminates. The convergence of experimental results and FE simulations validates the reliability of numerical models in predicting real-world behavior, reinforcing their critical role in designing composites for impact-critical applications, particularly in aerospace and industrial sectors.

### High velocity impact

The testing and validation of high-velocity impact (HVI) responses in composite materials such as carbon fiber composites, Kevlar-reinforced laminates, and textile-based body armor provide crucial insights into their ballistic performance.^167^^,^^168^^,^^208^ The HVI test setup typically includes a compressor, barrel, canister, and a sample-holding chamber, as illustrated in Figure 23D. The compressor is preset to achieve the desired bullet velocity, ensuring linear projectile motion through the barrel with canister support for accurate targeting. Key parameters influencing impact performance include bullet energy absorption, ballistic limits, areal density, bullet geometry, damage area, and the correlation between experimental results, finite element (FE) models, and analytical predictions.^209^ Tables 4 and 5 presents work done during penetration for different shaped bullet and HVI performance across various stacking sequences at different energy levels.

**Table 4:** Work done during penetration for different bullet shape

Bullet shape its parameters	Spherical	Conical Bullet	Flat-Nosed Bullet	Hollow-Point Bullet	Ogival (Pointed but Curved)
Contact area A(x)	πR^2^	π $R^{2}\frac{x}{h}$	πR^2^	π(R + kx)^2^	$\pi r^{2}\;\sin^{2}\left(\frac{x}{R}\right)$
Resistive force: F(x)	σ_y_πR^2^	$\sigma_{y}\pi R^{2}\frac{x}{h}$	σ_y_πrR^2^	σ_y_π(R + kx)^2^	$\sigma_{y}\pi r^{2}\;\sin^{2}\left(\frac{x}{R}\right)$
work done during penetration (W_p_)	σ_y_πR^2^d	$\frac{\sigma_{y}\pi R^{2}}{2h}d^{2}$	σ_y_πR^2^d	$\sigma_{y}\pi \left[R^{2}d+2\text{Rk}d^{2}+\frac{k^{2}d^{3}}{3}\right]$	$\frac{\sigma_{y}\pi r^{2}R}{2}\left[d-\frac{R}{2}\;\sin\left(\frac{2d}{R}\right)\right]$
Terminology	where “R” is the radius of the sphere and “d” is the penetration depth	where “R” is the base radius and “h” is the cone height.	where “R” is the base radius and “h” is the cone height.	where “k” is the expansion rate.	where “R” is the radius of the ogive.

**Table 5:** HVI ballistic performance of different laminates

Laminate Type	Ballistic Limit (m/s)	Area of Damage Predicted by NDT	Key Findings	Reference
Carbon Fiber Laminates	Varies with thickness	Mapped using CT and ultrasonic scans	Validated FE model against experimental results; effective in predicting damage mechanisms	English et al.^203^
Composite Plates	Varies with thickness	Good correlation with experimental damage areas	Developed global/local modeling approach; validated against multiple impact scenarios	Caputo et al.^204^
Kevlar® Helmet	610 m/s (FSP)	Indentation observed at the impact spot	Validated simulations against experimental results; effective in predicting damage mechanisms.	Tham et al.^210^
Kevlar®/PP Composites	376 m/s (2D-composites), 470 m/s (3D-composites)	Damage patterns consistent with NDT results	Hydrocode simulations showed good correlation with experimental results; 3D fabrics performed better than 2D.	Bandaru et al.^164^
Advanced Combat Helmet	610 m/s (FSP), 358 m/s (9-mm)	Mapped using CT and high-speed photography	Validated FE model against experimental results; effective in predicting damage mechanisms.	Palta et al.^211^
Armor-Grade Steel	365 m/s	Damage patterns consistent with simulations and NDT results	Numerical simulations showed good correlation with experimental results; MJC and CL criteria performed well.	Choudhary et al.^13^
Carbon Fiber Composites	–	Mapped using high-speed imaging	Validated FE model against experimental results; effective in predicting damage mechanisms	Chocron et al.^166^
Polymer Matrix Composites	300 m/s (2D), 270–280 m/s (3D)	Damage patterns consistent with simulations and NDT results	Generalized analytical model validated against experimental results; captures energy absorption mechanisms	Shaktivesh et al.^212^
Multi-Ply Woven Fabrics	190 m/s (3-ply), 310 m/s (10-ply)	Mapped using high-speed imaging	Validated multi-scale model against experimental results; effective in predicting damage mechanisms.	Palta, and Fang^213^
Fiber-Reinforced Composite/Metal	Varies with fiber configuration	Damage patterns consistent with simulations and NDT results	Experimental and numerical analysis showed good correlation; Kevlar® front plate provided better ballistic resistance.	Dong et al.^180^
AM355 Steel	350 m/s	Mapped using high-speed imaging	Validated FE model against experimental results; effective in predicting damage mechanisms	Zhang et al.^214^
Combat Helmet	697 m/s	Damage patterns consistent with simulations and NDT results	Numerical model validated against experimental results; good correlation in Back face deformation (BFD) predictions	Rodríguez-Millán et al.^181^
Kevlar® Fabrics	–	Mapped using high-speed imaging	Validated FE model against experimental results; effective in predicting damage mechanisms	Chocron et al.^215^
Multi-Layer Woven Fabrics	–	Damage patterns consistent with simulations and NDT results	Analytical model validated against experimental results; effective in estimating exit velocities	Chen et al.^216^
Kevlar® Soft Armour	126.55 m/s	Mapped using high-speed imaging	Validated FE model against experimental results; effective in predicting V_0_-V_100_ curve.	Nilakantan et al.^16^
Hybrid 3D Orthogonal Woven Composite	Effective resistance against conical and cylindrical projectiles	Damage patterns consistent with simulations and NDT results	Multi-scale modeling approach validated against experimental results; good correlation in energy absorption.	Dewangan, and Panigrahi^217^
B4C Composite Armour	710 m/s (flat), 748 m/s (sharp)	Mapped using high-speed imaging	Validated FE model against experimental results; effective in predicting damage mechanisms.	Zhang et al.^218^
Blunt Ballistic Impacts	–	Injury patterns analyzed by NDT	Established design and injury criteria for blunt ballistic impacts; validated using cadaver data.	Bir, and Viano^219^
Kevlar® Composites	–	Mapped using SEM	Validated FE model against experimental results; FGK showed better impact resistance due to Al_2_O_3_.	Degnah et al.^220^
3D Orthogonal Woven Composites	Effective resistance against conical-cylindrical projectiles	Damage patterns consistent with simulations and NDT results	Multi-scale modeling approach validated against experimental results; good correlation in energy absorption.	Dewangan, and Panigrahi^221^

**Figure 24:**
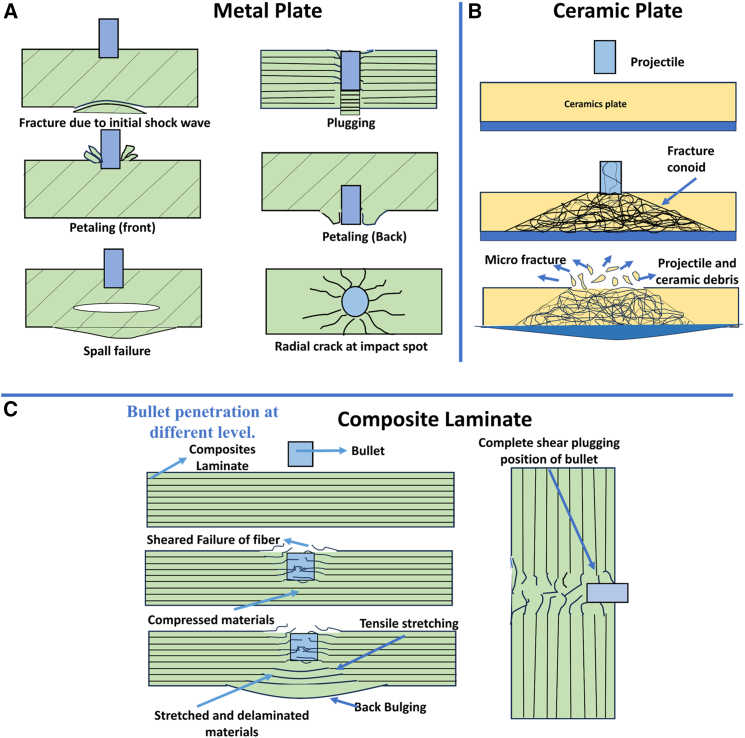
HVI damage mechanisms on metal, ceramic and composite plates (A–C) Characteristic high-velocity impact damage mechanisms: (A) metals petaling/spalling, (B) ceramics fragmentation and plugging, and (C) composites shear plugging, fiber rupture, matrix cracking, and back-face bulging; photographic/CT examples included.

**Figure 25:**
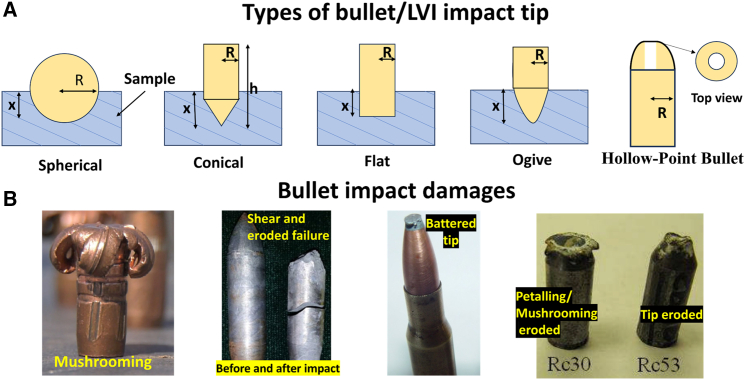
Projectile shapes and HVI impact damage examples (A) Representative projectile shapes considered (flat, conical, sharp) for LVI and HVI testing, and (B) examples of projectile-induced damage modes observed after high-velocity impact. Refs.^223^^,^^224^

**Figure 26:**
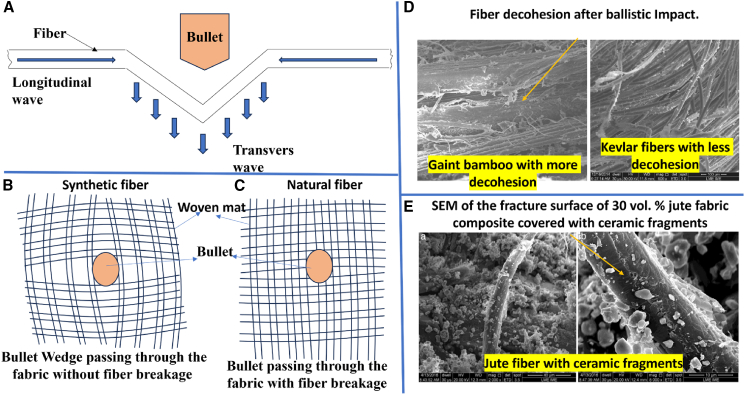
Fiber response and fragment entrapment during HVI (A–C) High-tensile synthetic fiber response under HVI (bullets wedge through fabric with limited fiber breakage; yarn stretching), (D and E) comparison images showing limited decohesion in Kevlar vs. enhanced fragment entrapment and microfibrillation observed in selected natural fibers (e.g., giant bamboo, jute). Data and criteria compiled from Refs.^225^^,^^226^

Figures 24A and 24B illustrate typical metal failure modes such as petaling, spalling, plugging, and radial cracking caused by reflected tensile shock waves during impact.^222^ In ceramics, high brittleness leads to conoidal fractures at the impact site, with crack propagation occurring through transgranular and intergranular modes, resulting in pulverized debris.^218^ Due to their high hardness and fracture toughness, ceramic plates absorb significant kinetic energy and induce projectile damage mechanisms such as petaling, mushrooming, shear, erosion, and tip deformation (Figure 25B). The initial compressive load from the bullet is transferred to the fiber and transformed into longitudinal and transverse shock waves as demonstrated in Figure 26A, which induce in-plane shear and out-of-plane tensile stresses that promote plate shearing. The transverse shock wave velocity (u) is a function of longitudinal wave velocity and fiber strain (*ε*) given in the Equation 34. Owing to their high elastic modulus, tensile strength, failure strain, and low density, natural fibers are emerging as viable alternatives to synthetic fibers in armor applications.

(Equation 34) $$u=c\sqrt{\frac{\varepsilon }{1+\varepsilon }}$$

Cunniff^85^^,^^227^ formulated the dimensionless fiber property (η) as a combination of specific fiber toughness and strain wave velocity given in the Equation 35, aiding in the design of armor systems with optimal bullet-resistant characteristics. More specifically, natural fiber possesses potentially equal vent specific fiber toughness and higher strain with lower density compared to synthetic fibers, as demonstrated in Figures 11 and 12.

(Equation 35) $$\eta =\frac{\sigma \varepsilon }{2\rho }c$$

Where “σ” denotes the ultimate tensile strength of the fiber, yarn, or fabric (Pa), “ε” represents the material’s strain, “ρ” is the yarn or fiber bulk density $\left(\frac{Kg}{m^{3}}\right)$ and “c” signifies the longitudinal wave velocity $\left(\frac{m}{s}\right)$.

Bullet energy absorption is a key metric for evaluating the protective capabilities of composite materials. English et al.^203^ demonstrated that carbon-fiber laminates absorbed a significant amount of energy during high-velocity impacts, with 23 J absorbed out of a 50 J impact, which contributed to mitigating damage and maintaining structural integrity. Similarly, Chocron et al.^215^ and Nilakantan et al.^16^ highlighted that the structural properties of fabrics, such as yarn linear density and fabric thread density, play a crucial role in energy absorption. Chocron et al.^215^ modeled the ballistic impact of various projectiles on Kevlar, finding that fabric structure significantly influenced energy dissipation during impact. Nilakantan et al.^16^ used FE simulations to predict energy absorption in Kevlar soft armor, showing a close correlation between the fabric properties and the absorbed energy, particularly with varying projectile velocities. Moreover, Dewangan and Panigrahi^217^ demonstrated that hybrid 3D orthogonal woven composites exhibited increased energy absorption with higher impact velocities. This highlights the role of material design, including fiber architecture, in optimizing energy dissipation for ballistic protection.

Composite materials also demonstrate distinct energy absorption mechanisms. Zhang et al.^218^ noted that boron carbide (B_4_C) composite armor dissipated energy via fragmentation of the ceramic panel and absorption by the UHMW-PE back plate. The addition of Al_2_O_3_ to functionally graded Kevlar composites (FGK) enhanced their energy absorption by 31.8% compared to regular Kevlar composites (KCM).^220^ These studies illustrate that both material composition and structural design play pivotal roles in energy absorption under ballistic impacts.

Ballistic limit is defined as the minimum velocity at which a projectile can penetrate a material, providing a critical measure of material performance under impact conditions. English et al.^203^ observed that thicker laminates with optimized fiber orientations achieved higher ballistic limits in composite materials. In a similar vein, Tham et al.^210^ found that Kevlar helmets exhibited a ballistic limit of 610 m/s against fragment-simulating projectiles. Bandaru et al.^164^ noted that 3D Kevlar laminates achieved higher ballistic limits (470 m/s) compared to 2D laminates (376 m/s), underlining the influence of advanced weaving techniques on ballistic resistance. Additionally, Chocron et al.^166^ and Shaktivesh et al.^212^ found that ballistic limits in carbon fiber composites also increased with laminate thickness, aligning with numerical predictions. The relationship between laminate thickness, fiber orientation, and ballistic limit emphasizes the need for optimized material configurations to enhance protective performance.

Areal density, which represents the weight per unit area, significantly affects both the weight and energy absorption capabilities of composite materials. English et al.^203^ and Palta et al.^211^ emphasized the importance of optimizing areal density to balance lightweight characteristics with sufficient energy absorption. This optimization is critical in applications such as body armor, where reducing weight while maintaining high energy absorption is essential. The bullet shape also plays a crucial role in penetration dynamics and damage patterns. Chocron et al.^215^ and Dewangan and Panigrahi^217^ noted that different bullet shapes, such as cylindrical and conical projectiles, caused distinct failure mechanisms in fabrics and composites. Conical projectiles typically induced more localized damage, affecting penetration depth and the extent of damage. The variation in bullet shape affects how energy is transferred to the material and, consequently, its ability to absorb impact.

The areal density of armor materials significantly affects their performance. Optimizing areal density in Kevlar fabrics enhances energy absorption while maintaining lightweight characteristics.^16^^,^^215^ Similarly, B_4_C and functionally graded Kevlar (FGK) composites exhibited higher ballistic resistance due to optimized areal densities.^218^^,^^220^

Because the shape of the bullet affects the distribution of the resistive force (F) and penetration depth (d), mathematical equations for penetration work are dependent on the bullet’s shape. The equations for various bullet forms based on penetration mechanics is given as follows. The work done during penetration (*W*_*p*_) for different bullet shapes is given in Table 4.

(Equation 36) $$W_{p}=\int_{0}^{d}F\left(x\right)\;d\left(x\right)$$

During high-velocity impact, flat-nosed bullets require the most work for penetration due to their large surface area and high resistance, while hollow-point bullets dissipate energy but need slightly less work. Spherical bullets cause moderate work, and conical or ogival bullets are the most efficient, requiring the least work due to their streamlined design.^52^ The work done during penetration at HVI for different bullet shapes was given analytically in Table 4 and demonstrated in Figure 24A. Chocron et al.^228^ found experimentally that blunt projectiles cause extensive delamination and shear plugging, while sharp-nosed ones produce cleaner penetration with less peripheral damage; oblique impacts lead to asymmetric damage and altered back-face deformation.

The area of damage resulting from high-velocity impacts is a critical metric for evaluating the effectiveness of composite materials in protective applications. High-speed imaging and diagnostic tools are widely utilized to investigate how impact energy, bullet geometry, and material properties influence damage patterns. Tham et al.^210^ reported surface indentation with minimal delamination in Kevlar helmets, while English et al.^203^ employed ultrasonic scans and CT imaging to correlate damage extent with bullet shape and energy. Chocron et al.^215^ and Chen et al.^216^ demonstrated that both projectile velocity and type significantly influenced damage patterns, as captured through high-speed imaging. Complementary finite element (FE) simulations by Nilakantan et al.^16^ and Dewangan and Panigrahi^217^^,^^221^ supported these experimental observations. Furthermore, SEM analysis by Degnah et al.^220^ revealed semi-bridge structures in functionally graded Kevlar (FGK) composites that enhanced load transfer efficiency and reduced damage spread. Collectively, these studies confirmed that the damage area increases with impact energy and varies with projectile type, underscoring the need to optimize material design for minimal damage and maximum energy absorption.

Distinct from the damage in metal and ceramic plates under HVI, which typically involves petaling, spalling, and plugging fiber-reinforced polymer composites (FRPCs) exhibit shear plugging, fiber failure, stretching, delamination, and back-face bulging, as shown in Figure 25C. FRPCs offer greater potential for enhancing ballistic performance through mechanisms such as fiber rupture and matrix-fiber decohesion. Due to the high tensile strength of synthetic fibers under high strain rate loading, bullets can often wedge through the fabric without breaking fibers, as illustrated in Figures 26A–26C. In contrast, natural fiber composites are more likely to undergo fiber breakage along with effective tensile stretching, contributing to better kinetic energy dissipation. Additionally, the helicoidal microstructure of the cellulose-rich outer layers in natural fibers facilitates shock-induced decohesion into thin microfibrils during impact, which enhances their capacity to trap and dissipate high-temperature ceramic and metal fragments from multi-layered armor systems (MAS). Figures 26D and 26E highlight that Kevlar fibers show limited decohesion and fragment entrapment compared to natural fibers such as giant bamboo and jute,^225^^,^^226^ emphasizing the superior fragmentation control offered by certain natural fiber systems.

The findings from experimental testing have been further validated through finite element (FE) simulations and analytical models, which have proven effective in predicting the impact responses and damage evolution in composite materials. Bandaru et al.^164^ and Chocron et al.^166^ employed advanced FE simulations (using ANSYS AUTODYN and LS-DYNA) to predict damage patterns under ballistic impacts, with good agreement between simulated and experimental results. These models, when combined with experimental validation, offer valuable insights into optimizing laminate configurations for improved impact resistance. Furthermore, Chocron et al.^215^ and Chen et al.^216^ used analytical models that successfully predicted the ballistic limits and energy absorption behaviors of multi-layered fabric and composite systems, providing a robust framework for material design. Similarly, Zhang et al.^218^ and Degnah et al.^220^ demonstrated that FE models could effectively simulate the energy absorption and damage mechanisms in B4C and FGK composites. Multi-scale modeling approaches by Dewangan and Panigrahi^217^ provided detailed analyses of hybrid 3D woven composites, confirming the models’ utility in optimizing material performance. In terms of damage, unidirectional laminates primarily fail through fiber rupture, splitting, and localized matrix shear, offering limited crack-arrest capability due to their aligned fiber paths. Helicoidal laminates, in contrast, exhibit progressive ply-by-ply fragmentation, enhanced crack deflection, and broader delamination zones, which improve through-thickness shear resistance. Hybrid laminates combine the distinct failure characteristics of their constituent fibers, enabling staged energy dissipation. High-modulus fibers counter initial impact, while ductile or natural fibers absorb post-impact deformation through mechanisms such as pull-out and fibrillation.

In conclusion, the combination of experimental testing and finite element modeling has significantly advanced our understanding of high-velocity impact performance in composite materials and textile-based body armor. Studies consistently emphasize the importance of optimizing energy absorption, ballistic limits, areal density, and damage area for improved material performance. These findings highlight the critical role of material architecture, fiber orientation, and advanced weaving techniques in enhancing ballistic resistance and minimizing damage. As the field continues to evolve, integrating experimental data with predictive models remains essential for developing next-generation materials for high-performance ballistic applications.

## Standards and compliance

ASTM standards play a crucial role in the evaluation of ballistic and blast performance of armor systems, ensuring the effectiveness and reliability of protective gear across various applications. This section elaborately discusses the standards followed to evaluate the ballistic performance of fiber composites.

ASTM D3776,^229^ provides standard methods for determining the mass per unit area of fabrics, a key metric in assessing the weight and comfort of soft body armor.^230^ ASTM F1342,^231^ defines the test method for evaluating the ballistic resistance of personal body armor, ensuring compliance with performance criteria for protection against diverse ballistic threats. For vehicle armor systems, ASTM F2656,^66^ sets the standard for assessing ballistic resistance, vital for military and law enforcement vehicle protection. Together, these standards offer manufacturers and users the assurance that protective equipment meets rigorous safety and performance benchmarks.^44^^,^^230^ Ballistic standards applicable to high strain rate testing are summarized in Table 6.

**Table 6:** Different ballistic standards are followed for high strain rate experiments

Test Type	Standard	Reference
Low-Velocity Impact	ASTM D7136	^232^
Compression after Low-Velocity Impact, SHPB compression	ASTM D7137	^233^
Tensile after Low-Velocity Impact, SHPB tensile	ASTM D638	^234^
Bending after Low-Velocity Impact	ASTM D7914	^235^
SHPB shear	ASTM D3518, D7078	^232^^,^^236^
Accessory ballistic panels, Body armor, Hard armor or rigid armor,	NIJ-0101.06	^19^
Ballistic limit, Ceramic composite armor, Composite armor	MIL-STD-662F	^237^
Armour panel (e.g., the front and back panels), Back-face signature, Backing material (clay withness), Baseline ballistic limit	NIJ-0101.04/06	^19^

For evaluating composite materials under impact, particularly fiber-reinforced polymer matrix composites, ASTM D7136,^233^ and ASTM D7137,^233^ are essential. ASTM D7136 specifies a drop-weight impact test to assess low-velocity impact resistance particularly relevant to aerospace and automotive industries.^198^^,^^207^ Complementing this, ASTM D7137 provides the methodology for measuring compressive residual strength after impact, thus offering insights into structural integrity under post-impact service conditions.^50^^,^^202^

Beyond ASTM, other ballistic standards such as NIJ and MIL-STD-662F provide comprehensive guidelines for evaluating armor systems. The NIJ Standard-0101.06 and MIL-STD-662F,^19^^,^^238^ define performance benchmarks for both flexible and hard body armor. Flexible armor,^19^ composed of textile materials, provides mobility with handgun protection, while hard armor featuring rigid inserts offers enhanced protection against rifle threats, either standalone or as part of in-conjunction systems. In-conjunction armor combines both flexible and rigid components, necessitating integrated testing at both component and system levels to meet designated threat criteria.

Fragment-simulating projectiles are employed to assess protection against explosive threats, while standardized full-metal-jacketed (FMJ) bullets comprising lead cores with copper alloy jackets are specified in NIJ-0101.04/06 and NIJ-0101.08,^19^ Other design parameters include obliquity (impact angle), overmatch and undermatch conditions (relative projectile and armor sizing), and damage metrics such as penetration, perforation, and back-face signature (BSF). As per NIJ-0101.06, a BSF deformation greater than 44 mm is unacceptable. Terminal ballistic assessments involve projectile behavior such as fragmentation and penetration, while V50 testing identifies the velocity at which 50% of projectiles are expected to perforate the armor. Additional parameters such as witness plates for spall detection, projectile yaw, and wear-face orientation further enhance the reliability and uniformity of armor evaluation. Table 7 summarizes testing criteria per NIJ-0101.06 for different gun types, including the number of hits per panel, bullet weights, and their corresponding velocities. Current ASTM, NIJ, and MIL standards are optimized for synthetic composites, making it challenging for sustainable FRPCs often sensitive to moisture and property variability, to qualify. Bridging this gap requires both material innovations and evolving standards that also account for sustainability metrics such as recyclability and carbon footprint.

**Table 7:** Summary of ballistic limit and energy absorption of reported composite systems

NIJ Protection Level	Ammunition Type	Bullet Weight (g)	Bullet Velocity (m/s)	Protection Against	Number of hit per panel
Level IIA	9 mm FMJ RN	8.0	373	Low-velocity handgun rounds (9 mm, 0.40 S&W)	5
	0.40 S&W FMJ	11.7	352		
Level II	9 mm FMJ RN	8.0	398	Standard handgun rounds (0.357 Magnum, 9 mm)	
	0.357 Magnum JSP	10.2	436		
Level IIIA	0.357 SIG FMJ FN	8.1	448	High-velocity handgun rounds (0.44 Magnum, 9 mm)	
	0.44 Magnum SJHP	15.6	436		
Level III	7.62 mm NATO FMJ (M80)	9.6	847	Rifle rounds (7.62 mm NATO, 0.308 Winchester)	
Level IV	0.30-06 M2 AP	10.8	878	Armor-piercing rifle rounds (0.30 caliber AP)	1
Special Threats	Customized testing	Varies	Varies	Protection against specific threats (e.g., 5.56 mm, AP rounds)	1

Compilation of ballistic limit velocities, energy absorption, and material configurations reported for various fiber-reinforced composite systems under high-velocity impact. Data summarized from Refs.^39^^,^^40^^,^^41^^,^^42^^,^^43^^,^^44^^,^^45^^,^^46^^,^^47^^,^^48^^,^^49^^,^^50^^,^^51^

## Impact damage and non-destructive testing techniques

In this section discussed on Non-destructive testing (NDT) techniques as emerged as indispensable tools in the characterization of damage in composite materials, particularly for understanding the influence of stacking sequences, fiber arrangements, and reinforcement architectures on damage propagation and impact performance.

**Figure 27:**
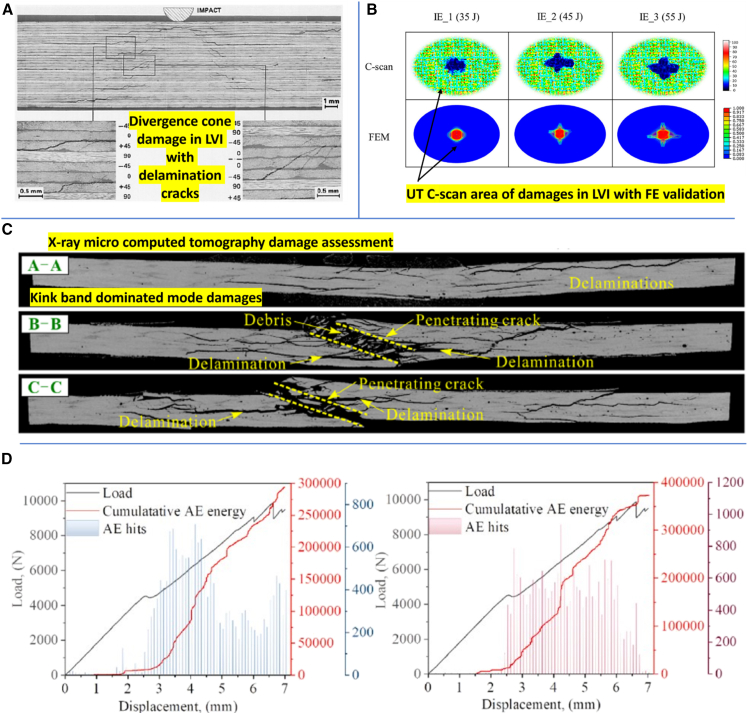
NDT visualizations of LVI damage and AE monitoring (A) Divergence-cone crack observed in thick laminate via X-ray CT, (B) C-scan ultrasonic surface/delamination map for carbon/glass laminates with FEM validation, (C) X-ray micro-CT of internal damage zones, and (D) acoustic emission (AE) hits and cumulative AE energy correlated with matrix cracking and delamination. Data and criteria compiled from Refs.^140^^,^^143^

Figures 27A–27C demonstrate divergence cone damage in LVI with delaminated crack through X-ray computed tomography (CT), surface area damages through ultrasonic C-scanning, and kind band dominated mode internal damages through X-ray micro computed tomography.^140^^,^^143^ Saleh et al.,^50^ employed ultrasonic C-scanning and CT to analyze internal damage in composites post-impact, demonstrating the capability of NDT to correlate internal damage mechanisms with stacking configurations and fiber orientations. Their work revealed that alternating fiber orientations exhibited enhanced damage tolerance and lower damage accumulation compared to unidirectional or sandwich-like configurations, providing critical insights for designing impact-resistant composites.

Ricciardi et al.,^137^ highlighted the utility of confocal microscopy in measuring indentation and assessing damage propagation after impact, while Jiang et al. utilized similar methods to evaluate internal delamination. Meanwhile, Wu et al.,^140^ employed X-ray micro-computed tomography (μ-CT) to visualize internal damage in braided laminates post-impact. Their analysis provided a deeper understanding of damage mechanisms, such as crack propagation and delamination, emphasizing the effectiveness of nonwoven reinforcements in inhibiting these failure modes. Similarly, Hadj-Djilani et al.,^141^ used X-ray tomography to visualize void content and internal damage in flax/epoxy composites, further illustrating the capability of μ-CT to evaluate damage characteristics comprehensively.

Advanced techniques such as ultrasonic C-scanning, profilometry, and fluorescent penetrant inspection (FPI) have proven effective in characterizing damage extent. Lyu et al.,^143^ used ultrasonic C-scanning to assess delamination and damage extent in hybrid laminates, while Sergi et al.,^131^ utilized profilometry to measure permanent indentation depth and analyze the damage. Lopes et al.,^134^ demonstrated the utility of FPI in comparing delamination sizes with numerical predictions, reinforcing the significance of NDT in bridging experimental observations and modeling results. Further, Bandaru et al.,^149^ employed high-definition digital imaging to analyze post-impact damage patterns, confirming that quasi-isotropic stacking sequences showed reduced damage compared to unidirectional and sandwich-like configurations.^151^

The integration of X-ray CT and surface scanning has also provided significant advancements in damage characterization. D. Chen et al.,^147^ used X-ray CT to assess delamination in hybrid laminates, while 3D surface scanning techniques were employed to measure deformation and damage areas on impacted specimens. These methods allowed for the detailed visualization of internal structures and highlighted the importance of stacking sequences in enhancing damage tolerance. The micro-CT analysis by Tiyek and Kaya^153^ provided insights into damage propagation in composites, confirming the effectiveness of nonwoven reinforcements in improving structural integrity.

NDT techniques have also proven essential in experimental and visual analysis of impact zones. Dye penetrant inspection, utilized by Pandian et al.,^155^ effectively highlighted damage areas in glass and jute fiber composites post-impact, offering a straightforward method for assessing damage zones. Similarly, ultrasonic C-scanning, as employed by Seyed Yaghoubi et al.,^239^ provided detailed internal damage assessments in GLARE 5 laminates, validating the influence of stacking sequences on damage patterns. Karimzadeh et al.,^240^ used scanning electron microscopy (SEM) to analyze fractured surfaces of PALF/glass fiber composites, shedding light on interfacial bonding and failure mechanisms. Ultrasonic testing method used to detect internal flaws and assess the extent of damage in composite materials.^73^

Beyond traditional methods, novel approaches such as infrared thermography, eddy current testing, and laser scanning have expanded the scope of NDT applications. Zhu et al.,^241^ demonstrated the utility of infrared thermography in assessing impact damage in CFRP laminates, highlighting its effectiveness in detecting subsurface anomalies and crack propagation. Rellinger et al.,^242^ showcased the potential of eddy current testing in locating impact sites in honeycomb sandwich panels, while laser scanning was integrated to detect deformations and irregularities. These studies underline the benefits of combining multiple NDT methods for comprehensive damage characterization. In particular, Rellinger et al.,^242^ found that integrating thermography, eddy current testing, and laser scanning effectively identified core crush, surface dents, and adhesive disbonds in aerospace composites. Swiderski^243^ demonstrated the efficacy of active infrared thermography in detecting ballistic impact damage in military-grade composites, further extending the applicability of NDT in high-performance applications.

The use of guided waves and thermography, as reported by Schmidt et al.,^244^ for monitoring fatigue damage in composite tubes provided valuable insights into the durability of impacted specimens. Their findings indicated reduced fatigue strength and service life for damaged composites, emphasizing the role of NDT in ensuring long-term reliability. Acoustic Emission (AE) is an effective non-destructive technique for real-time monitoring of damage in composites during impact.^245^ AE signal parameters such as amplitude, energy, and frequency help distinguish between damage modes such as matrix cracking, delamination, and fiber breakage. Figure 27D illustrates the AE hits and cumulative energy of damage modes under indentation, correlated with the load–displacement curve to reveal damage evolution. Advanced analyses such as wavelet packet decomposition enhance damage characterization. AE data, when correlated with mechanical responses and post-impact inspections, offers insights into damage progression and can be used to predict residual strength. Thus, AE plays a vital role in assessing the structural integrity of impacted composites.^246^

Collectively, these studies emphasize the versatility and indispensability of NDT methods, from ultrasonic and optical microscopy to advanced techniques such as AE, thermography, and μ-CT, in characterizing damage, optimizing design parameters, and enhancing the impact performance of composite materials. These methodologies have critical implications for industries such as aerospace, automotive, and civil engineering, where structural integrity and durability are paramount.

## Artificial intelligence in ballistic impact application

In this section discussed on Machine learning (ML) methods followed and emerged as a transformative tool in the analysis, prediction, and optimization of composite materials, spanning a wide range of applications from impact behavior to property prediction and design optimization.^55^

**Figure 28:**
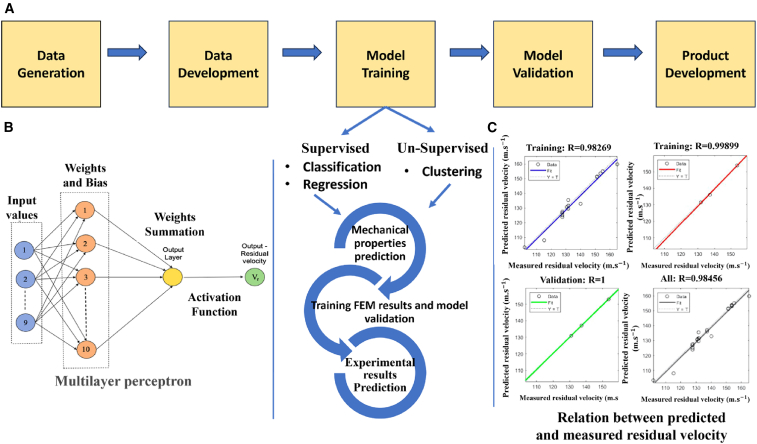
Machine-learning workflow and MLP validation for HVI prediction (A) ML workflow linking experimental/FE datasets to ML training and model deployment, (B) multilayer perceptron (MLP) architecture schematic (input, hidden layers, output), and (C) parity plot showing relation between predicted and measured HVI results (training/validation). Data and criteria compiled from Ref.^247^

The work flow of ML involves data generation and development form the experiments, model training through ML algorithm, The validation of results with FE or experimental results, and product development. ML algorithm is used for composite mechanical properties prediction for FEM validation or training FEM results to predict and validate experimental values. As demonstrated in Figure 28A, ML is used in a cyclic relation to FEM and experimental validation for an iterative and connected workflow to improve the precision, dependability, and effectiveness of predictions in ballistic impact. The extensive body of literature reviewed here highlights the diverse methodologies and outcomes achieved using ML techniques. Table 8 lists the numerous experiment kinds and applications for which the properties of composite materials are predicted using various machine learning techniques for distinct goals.

**Table 8:** Artificial Intelligence in ballistic impact application

Experimental Type	Materials Tested	Machine Learning Model	Key Finding from Machine Learning Methods	Reference
Design of reinforced polymer composites	Various reinforced polymer composites	Support Vector Machines (SVM), ANN, Hybrid	ML models significantly enhanced the identification of optimal material combinations and predicted properties.	Okafor et al.^57^
Impact resistance of composite armor	Multilayer composite armor (steel, ceramic, UHMWPE)	Hybrid model (SVM and Deep Neural Network)	Achieved rapid predictions of armor penetration and deformation, with high accuracy and reduced computation time.	Zhu et al.^248^
Machining accuracy of banana fiber composites	Banana fiber-reinforced epoxy composites	Ensemble (ANN and LSTM)	Achieved high predictive accuracy with R-squared values of 0.9758 (Ra), 0.9963 (MRR), and 0.9874 (Ka).	Saravanakumar et al.^249^
Low-velocity impact analysis of laminated composites	Laminated composite plates (carbon/epoxy)	ANN-PHFGMC	The ANN-PHFGMC model predicted nonlinear behavior with less than 5% error, effectively integrating micromechanics into FE analysis.	Hochster et al.^250^
Low-speed impact localization	Composite laminates (glass fiber reinforced epoxy)	RIME-optimized Dual-layer Support Vector Regression (RDSVR)	Achieved accurate impact localization with significant noise reduction and improved RMSE.	Shen et al.^64^
Projectile penetration prediction	Carbon/aramid hybrid fiber laminates	Neural Network, Decision Tree	Neural network model demonstrated higher predictive accuracy (R = 0.9979) compared to decision tree.	Wang, and Sun^251^
Impact damage assessment	Braided Carbon/Kevlar® tubes	Artificial Neural Network (ANN)	ANN models achieved an MSE of 0.000191 kN, accurately predicting load-bearing capacity post-impact.	Laban et al.^252^
Underwater explosion response prediction	Coated composite cylinders (polyurea and epoxy)	Feedforward Multilayer Perceptron (MLP)	MLP model achieved an R^2^ value of 0.85, effectively predicting the dynamic response of cylinders under UNDEX.	Nayak et al.^253^
Low-velocity impact damage assessment	Glass/Epoxy woven laminates	MLP, KNN, DT, SVR	Models estimated damage properties with 4%–6% accuracy compared to experimental results.	Jalali et al.^56^
Nanoindentation data analysis	Carbon Fiber Reinforced Polymers (CFRPs)	SVM, ANN, Classification Trees	SVM achieved 67% accuracy in classifying reinforcement mechanisms based on nanoindentation data.	Konstantopoulos et al.^254^
Ballistic performance analysis	Composite armor systems (steel, aluminum, MWCNTs)	Random Forest (RF)	High accuracy in predicting ballistic performance, with MWCNT-reinforced composites showing superior results.	Tsirogiannis et al.^40^
Impact behavior prediction	Carbon Fiber Reinforced Polymers (CFRPs)	Random Forest, Multi-output regression	Multi-output regression model achieved R^2^ > 0.995, accurately predicting impact curves for varying void contents.	Mendikute et al.^255^
Terminal ballistics prediction	Various projectile types and target materials	XGBoost, ANN, SVR, Gaussian Process (GP)	GP model demonstrated high predictive accuracy and uncertainty quantification across diverse projectile and target conditions.	Shen et al.^46^
Physical properties prediction	Textile polymer composite materials	ANN, SVM (optimized with MOPSO, NSGA-II, SPEA2)	Enhanced predictive accuracy for physical properties of textile polymer composites through optimization.	Malashin et al.^256^
Residual strength prediction	Carbon Fiber Reinforced Composites (CFRCs)	ANN (back-propagation)	ANN model predicted residual strength with less than 5% error compared to FEA results	Yang et al.^257^
Impact performance prediction	Fiber Reinforced Polymer Composites (hybrid and non-hybrid)	MLP (ANN)	ANN predicted impact performance with a maximum variation of 6.6% compared to FEA results, validating its accuracy.	Stephen et al.^247^
Erosion rate prediction	Uni-Directional Glass Fiber Reinforced Polymer Composites	ANN, Extreme Gradient Boosting (XGB)	XGB model achieved R^2^ of 0.976, outperforming ANN in predicting erosion rates based on multiple parameters.	Deliwala et al.^258^
Wear and thermal performance analysis	Glass Fiber-Epoxy Composites with Carbon Nanotube Fillers	Machine Learning Regression Models	Carbon nanofillers significantly improved wear resistance and thermal properties, with accurate predictions from ML models.	Vaddar et al.^259^
Impact damage detection	Fiber-Reinforced Composites (Kevlar®, Basalt, Hybrid)	LSTM, 1D-CNN, FFNN, 1D-CNN + GRU	LSTM model outperformed others in detecting impact damage with high accuracy and recall across different materials.	Duan et al.^260^
Review of AI/ML applications	High-performance fiber-reinforced polymer composites	ANN, SVM, Genetic Algorithms	Significant improvements in efficiency and accuracy in material property prediction and damage diagnosis.	Wang et al.^261^
Design and analysis of composite materials	Various composite materials	ANN	Identified challenges in accurately predicting nonlinear behaviors of composite materials.	Liu et al.^262^
Crashworthiness optimization	Polypropylene sandwich tubes	MLP, NSGA-II	Optimized design led to significant improvements in energy absorption and crashworthiness.	Ma et al.^263^
Impact response prediction	Composite pipes	Enhanced ANN, Jaya, E-Jaya	Improved prediction accuracy for displacement after low-velocity impacts using enhanced ANN techniques.	Ghandourah et al.^264^
Fracture toughness prediction	Bio-nano-composites	Decision Tree, Adaptive Boosting	High accuracy in predicting fracture toughness despite limited training data.	Daghigh et al.^41^
Dynamic properties prediction	Ultra-high-performance concrete (UHPC)	Case-Based Reasoning (CBR)	CBR system achieved 81.5% prediction accuracy for dynamic mechanical properties, reducing experimental needs.	Khosravani et al.^265^
Damage detection and classification	Polycarbonate infused with AEROSIL	U-Net, YOLOv8, Mask R-CNN	Mask R-CNN effectively detected minor and major damages, outperforming other models in damage assessment.	Qarssis et al.^266^
Dynamic strength analysis	Polypropylene-based composites	ANN, SVR, RF, XGB, KNN, Decision Tree	ANN achieved the highest prediction accuracy for dynamic strength, demonstrating ML’s effectiveness in material analysis.	Cai et al.^267^
Geometry optimization for dynamic tension	Composite materials	Multi-Layer Perceptron (MLP)	MLP predicted optimal specimen geometries for dynamic tension tests, significantly reducing testing time and costs.	Gomez Consarnau, and Whisler^268^
Impact localization	Carbon Fiber Reinforced Polymer (CFRP) plates	Convolutional Neural Network (CNN)	Achieved an average localization error of 10.2 mm, outperforming traditional ToA methods in accuracy.	Feng et al.^269^

The architecture of Multilayer Perceptron (MLP) involves assigning weights and biasing the input data as hidden layer, followed by weight summation through an activation function to predict the output results. The efficiency of output predicted results is analyzed through training and validation plots as demonstrated in Figures 28B and 28C. Jalali et al.,^56^ utilized four regression models, including MLP, K-Nearest Neighbors (KNN), Decision Tree (DT), and Support Vector Regression (SVR), to predict damage properties such as modulus, tensile strength, and compressive strength of glass/epoxy woven laminates based on low-velocity impact (LVI) responses. Their models achieved prediction accuracies within 4–6% of experimental results, demonstrating the efficacy of ML in dynamic property estimation. Similarly, Konstantopoulos et al.,^254^ leveraged Support Vector Machines (SVM), Artificial Neural Networks (ANN), and classification trees to analyze nanoindentation data from CFRPs. Their findings emphasized the potential of SVM with a radial kernel, which achieved a 67% accuracy, for understanding interfacial properties in composite systems.

Expanding on the design front, Okafor et al.,^57^ utilized hybrid models to enhance the design process of reinforced composites by rapidly identifying optimal material combinations and predicting mechanical properties. Zhu et al.,^248^ applied a hybrid SVM-Deep Neural Network (DNN) model for the ballistic performance prediction of multilayer composite armors, achieving rapid and accurate predictions compared to computationally intensive traditional methods. Similarly, Tsirogiannis et al.,^40^ employed Random Forest (RF) models to evaluate ballistic performance across different composite configurations, highlighting the superior resistance of composites reinforced with multi-walled carbon nanotubes (MWCNTs).

Saravanakumar et al.,^249^ explored ensemble ML techniques combining ANN and Long Short-Term Memory (LSTM) networks to predict machining outcomes such as surface roughness, material removal rate, and kerf angle in abrasive water jet machining (AWJM). Their ensemble model demonstrated remarkable predictive accuracy with R-squared values above 0.97. Hochster et al.,^250^ developed ANN-based micromechanical modeling frameworks, achieving less than 5% error in predicting the nonlinear behavior of fiber-reinforced polymer composites under impact conditions.

The application of ML in predictive modeling has also been demonstrated in innovative ways. Shen et al.,^45^ integrated Adaptive Sparse Noise Reduction Algorithms (ASNRA) with RIME-optimized Dual-layer Support Vector Regression (RDSVR) for noise reduction and impact localization in composites. Wang and Sun^251^ showed that neural networks outperformed decision tree models in predicting projectile residual velocities, particularly in scenarios with limited sample sizes. Similarly, Laban et al.,^252^ trained ANN models to predict the axial compression capacity of pre-impacted composite tubes, achieving high accuracy with minimal mean squared errors.

In dynamic and ballistic applications, Nayak et al.,^253^ employed feedforward MLP models to predict the underwater explosion response of coated composite cylinders, achieving R^2^ values of 0.85. Mendikute et al.,^255^ used RF and regression techniques for impact behavior predictions of CFRPs, achieving R^2^ values exceeding 0.995. Ryan et al.,^46^ evaluated various models, including XGBoost and Gaussian Process Regression (GP), for predicting ballistic limits, with the GP model excelling in uncertainty quantification.

Optimization methods for ML models in composite applications were highlighted by Malashin et al.,^256^ who employed optimization algorithms such as MOPSO and NSGA-II for tuning ML hyperparameters. These optimized models improved the predictive accuracy of composite properties, as seen in studies such as Yang et al.,^257^ where ANN models predicted post-impact strength with less than 5% error.

The potential of deep learning was further showcased in studies such as Duan et al.,^260^ where Long Short-Term Memory (LSTM) networks detected impact damage in fiber-reinforced composites with high accuracy. Feng et al.,^269^ applied convolutional neural networks (CNNs) to localize low-velocity impacts on CFRP plates, achieving superior performance compared to traditional time-of-arrival methods.

In the context of erosion and wear analysis, Deliwala et al.,^258^ demonstrated the robustness of Extreme Gradient Boosting (XGB) over ANN in predicting erosion rates of glass fiber composites. Vaddar et al.,^259^ explored the influence of carbon nanotube fillers on composite performance using ML, showing accurate wear rate predictions. Cai et al.,^267^ applied ML models to 3D-printed polypropylene composites, with ANN achieving the highest prediction accuracy for dynamic strength.

Applications in structural monitoring and damage classification were reviewed by Qarssis et al.,^266^ who employed deep learning models such as Mask R-CNN for real-time damage detection in polycarbonate composites. Ghandourah et al.,^264^ enhanced ANN models with the Jaya algorithm to predict low-velocity impact displacements in composite pipes, showcasing improved prediction reliability.

Studies such as Khosravani et al.,^265^ highlighted alternative ML approaches, employing case-based reasoning systems to predict ultra-high-performance concrete properties. Daghigh et al.,^41^ used adaptive boosting and decision trees to predict bio-nano-composite fracture toughness, proving effective even with limited datasets. The reviewed works collectively emphasize the transformative role of machine learning in advancing composite material research and applications. By enabling accurate, efficient, and robust predictions, ML facilitates the design, analysis, and optimization of composites, driving innovations across industries. Key research gaps include developing FEA-ML hybrid models, multi-modal learning combining test data and imaging, standardized ballistic ML benchmarks, explainable AI for linking predictions to failure modes, and lifecycle-aware ML integrating sustainability metrics. Bridging ML’s adaptability with FEA’s physics-based rigor can enable real-time optimization, cost-effective design, and sustainable innovation in ballistic composites.

## Conclusion and future directions

Recent advances in FRPCs highlight how fiber architecture, matrix selection, hybridization, and stacking configurations govern performance under high strain rates and ballistic loading. Studies on unidirectional, quasi-isotropic, and helicoidal laminates demonstrate improved impact resistance, while combining natural fibers such as flax, hemp, and jute with aramid, basalt, or carbon enhances sustainability without compromising strength. Viscoelastic matrices such as polyurethane increase strain-rate sensitivity, energy dissipation, and damage tolerance. Integrated approaches using multi-criteria decision-making, finite element analysis, and non-destructive testing provide a data-driven framework for design optimization and quality assurance, complemented by machine learning for predictive modeling and damage classification. These strategies yield beneficial outcomes, including higher ballistic limits, reduced back-face deformation, and improved resilience for defense, aerospace, automotive, and civil protection. Future efforts should emphasize bio-based hybrids, advanced 3D textile architectures, ML-FEA integration, and circular economy principles to enable sustainable, multifunctional protective systems.

## Acknowledgments

The authors of this review article show their gratitude toward the institute and are greatly thankful to the Department of Metallurgical and Materials Engineering (MME), the 10.13039/501100008788National Institute of Technology Karnataka (NITK), Surathkal, Mangaluru, India, for allowing the resources to carry out this research. This research budget was allocated by National Science, Research and Innovation Fund (NSRF) (Fundamental Fund 2026), and King Mongkut’s University of Technology North Bangkok (Project no. KMUTNB-FF-69-A-03).

## Declaration of interests

The authors declared no potential conflicts of interest with respect to the research, authorship, and/or publication of this article.

## Supplemental information

Document S1. Tables A1–A3
