# Supplementary material for: Review on fiber composites for sustainable high strain rate applications
Source: iScience. 2025 Sep 19;28(11):113598. doi: 10.1016/j.isci.2025.113598 (PMC12590021; doi:10.1016/j.isci.2025.113598)
Supplement: Document S1. Tables A1–A3 [file mmc1.pdf]

iScience, Volume *28*

Supplemental information

Review on fiber composites for sustainable high strain rate applications

Darshan Madhapura Lakshme Gowda, Ravi Shankar Bhat, Sanjay Mavinkere Rangappa, and Suchart Siengchin

Supplementary Material

Appendix A. Supplementary tables

Table A1

Stacking configuration effect on SHPB properties.

Laminates types	SHPB Test	Stacking Types	Influence on properties	Reference
Glass/Epoxy	Compression load	Unidirectional balanced symmetric loaded in out-plane direction	High strain rate response; compressive strength sensitive to strain rate	[1]
Carbon Fiber/Epoxy (T700/BA9916)	Compression load	Unidirectional laminate $[0°]_{36}$ at In-plane loading	Exhibits significant temperature softening and strain-rate enhancement; compression failure strength increases with strain rate	[2]
Aramid Fabric Reinforced Polyamide	Compression load	Plain weave alternating layers of aramid and polyamide	High impact resistance; peak stress and strain increase with strain rate; suitable for protective application	[3]

Hybrid Kevlar®/Basalt/Polypropylene Composites	Compression load	Intra-ply and Inter-ply of Kevlar® and basalt laminate	Intra-ply hybrids show higher peak stress; inter-ply hybrids exhibit better strain rate sensitivity	4
E-Glass/Vinylester laminate ($[0°/90°]_{36}$)	Compression load	Woven 2D and 3D (stitching)	Enhanced compressive strength; damage mechanisms include fiber buckling and delamination	5
Polyamide 6/glass Composites	Compression load	Cross-ply fabric allied longitudinal and transverse direction to the load	Strain rate sensitivity observed; failure stress increases significantly with strain rate	6
Hybrid Carbon/Epoxy	Compression load	Cross-Ply laminate with nano particle reinforcement [0°/90°]n	Enhanced toughness; failure modes include delamination and fiber breakage.	7
Baslat/hemp/PU rubber composites Laminate	Compression load	Helicoidal laminate	Enhanced interlaminar shear strength; mitigated buckling and delamination	8

			failures; preferred for ballistic applications.	
Hybrid Composites polyethylene/ Aramid/ Dyneema woven fabric	Compression load	Various hybrid configurations	Improved energy absorption; L3 showed the highest energy absorption at all pressures.	9
Hemp, hemp/glass hybrid, cellulose, and wheat straw-reinforced polymeric composites	Compression load	Hybrid fiber laminates	Natural fibers dissipate energy at lower stress and higher strain compared to glass-reinforced composites.	10
CFRP Single Lap Joints	Tensile load	Various configurations joints	Increased joint stiffness and peak load with loading rate; energy absorption improves with higher rates.	11
Carbon Fiber/Epoxy Composites	Tensile load	0° and ±45° fiber orientations	Transition from fiber breakage and pull-out to brittle matrix cracking with increasing loading rates; tensile strength increases significantly.	12

Natural Fiber Reinforced Composites	Dynamic compressive properties	Unidirectional pultruded composites	Strain rate sensitivity observed; dynamic properties improve with increasing strain rates.	13
basalt–carbon/epoxy	V-notched rail shear test	Hybrid laminate	Stacking sequence affects shear strength significantly, with carbon fibers on the outer layers improving strength by 6.6% compared to basalt on the outer layers.	14
Carbon Fiber Reinforced Polymer (CFRP)	Shear load	unidirectional laminated at in-plane shear load	The shear strength and modulus increase with strain rate. The stacking sequence influences the shear properties, with higher strain rates leading to increased shear strength and modulus.	15

TableA2

Stacking configuration effect on LVI properties.

**Laminates types**	**Laminate structure types**	**Stacking Types**	**Influence on properties**	**Reference**
Graphite/Epoxy (AS4/3501-6)	Cross-Ply Laminates	$[90/0]_{6S}$	Higher delamination threshold load (DTL) and damage resistance with increased thickness.	16

Carbon Fiber Composite	Quasi-Isotropic Laminates	$[+45°/-45°/0°/90°]_{3S}$	Improved energy absorption and damage resistance; matrix cracking and delamination are critical.	17
IM7/8552 unidirectional carbon fibre pre-preg sheet	Cross-Ply Laminates	Various configurations	Stacking sequence affects the extent of internal damage; BVID can lead to significant structural issues.	18
Carbon Fiber Reinforced Polymer (CFRP)	Cross-Ply Laminates	$[0/90]_{6S}$	Improved damage tolerance and energy absorption; higher delamination threshold loads.	19
CFRP	Bio-inspired Helicoidal Composites (Symmetrical, balanced, non-symmetrical)	$[0/12/24/36/. . ./180]_{s}$	Enhanced damage resistance and impact energy absorption; lower through-thickness failure.	20
Carbon/Epoxy (T700GC/M21)	Symmetric	[0/90]2S, $[45°/0°/90°]_{3S}$	Improved damage tolerance and energy absorption; stacking sequence affects delamination behavior.	21

hybrid 3D woven composite made from S2-glass (GF), T700 carbon (CF) and Dyneema fibres.	3D Woven Composite	-	Balanced properties; good overall damage resistance but higher delamination compared to hybrid designs.	22
Carbon Fiber Reinforced Polymer (CFRP)	Cross-Ply	[0°/90°]	Improved damage tolerance; higher delamination threshold loads (DTL) compared to UD laminates.	23
Carbon Fiber Reinforced Polymer (CFRP)	Hybrid	[0°/±45°/90°]	Enhanced energy absorption and damage tolerance; stacking sequence affects failure mechanisms.	24
Carbon Fiber Reinforced Polymer (CFRP)	3D Woven Composite	Various configurations	Improved impact resistance; 3D architecture enhances energy absorption.	25
Carbon Fiber Reinforced Polymer (CFRP)	Laminated Composite	[0°/45°/-45°/90°]	Balanced properties; good overall damage resistance but higher delamination compared to hybrid designs.	23

Carbon Fiber Reinforced Polymer (CFRP)	Pulsed Infusion Unidirectional Laminates	$[(0)/(90)/(\pm 45)]_s$	Enhanced mechanical properties; reduced void content improves impact resistance.	24
Woven Kenaf Fiber Reinforced Composite	Hybrid composite laminate	[0°/0°/0°], [0°/15°/0°], [0°/30°/0°], [0°/45°/0°]	Higher impact strength at lower velocities; sensitivity to impact velocity; increased damage area around perforations.	26
Woven Jute Fiber Reinforced Composite	Helicoidal laminate	[0°/θ/0°] (θ varies from 0° to 45°)	Improved impact resistance with increasing fiber orientation; significant peak forces observed at different velocities.	27
Hybrid E-Glass Composites	Inter-ply hybrid laminates	Sandwich-like (SL) and Intercalation (IC)	SL sequences exhibited higher peak forces; IC sequences showed better energy absorption; hybridization improved overall impact resistance.	28
Sisal Fiber Reinforced Bio-composite	Bio-composites	Unidirectional (UD), Cross-ply (CP), Quasi-isotropic (QI)	Angle-ply laminates exhibited superior impact performance compared to other	29

			configurations; increased fiber volume fraction improved energy absorption.	
Woven Jute-Glass Hybrid Composite	Hybrid	Hybrid stacking (varying proportions of jute and glass)	Jute laminates showed better energy absorption than jute-glass hybrids; hybrids exhibited better damage tolerance.	30
Flax/PLA Bio-composite	Hybrid	Alternating layers of flax woven fibers and PLA	Exhibited non-linear mechanical behavior; strain rate significantly influenced stiffness and strength; damage primarily through fiber failure rather than delamination.	31

Table A3

FEM methods followed for high-strain rate experiments.

Materials and Test	Materials model considered	Test Type	Damage model considered for analysis	Properties compared with experimental results	Software package	Reference
woven E-glass/vinyl ester laminate	three-dimensional numerical model (which create orthotropic mechanical	SHPB compressive	Numerical models without damage were developed and successfully predicted the elastic behaviour of the materials	Strain, Velocity and Load	ABAQUS/Explicit	[5]

	properties in the three orthogonal directions)					
Carbon/epoxy composites	Mat-059-Composite Failure Model	SHPB compressive	cohesion zone model (CZM)	Strain and strain rate	ANSYS/LS-DYNA software.	2
Carbon Fiber Reinforced Aluminium Laminates (CARALLs)	Hashin method	SHPB Tensile	Hashin method	stress-strain curves.	Hyper-mesh	32
3D Fiber Metal Laminates (FMLs)	Continuum damage model	Low-velocity impact tests	Intraply and interply damage models, including delamination	Peak force, damage area, absorbed energy	ABAQUS	33

Bio-inspired Helicoidal Composites	User-defined subroutine (VUMAT)	Low-velocity impact tests	Cohesive damage model for delamination, intraply damage model	Peak force, delamination area, energy absorption	ABAQUS	[34]
Hybrid and Braided Laminates	Continuum damage model	Low-velocity impact and CAI tests	Intraply and interply damage models, including delamination	Residual compressive strength, damage area	ABAQUS	[35]
Dispersed Stacking Sequence Laminates	Continuum damage model	Low-velocity impact tests	Cohesive damage model for delamination, intraply damage model	Peak force, damage area, energy absorption	ABAQUS	[36]
Interlayer Hybrid Composites	Continuum damage model	Low-velocity impact tests	Matrix cracking, fiber breakage, and delamination models	Peak force, damage area, absorbed energy	ABAQUS	[37]
Thermoplastic Hybrid Composites	Linear-orthotropic damage model	Low-velocity impact tests	Matrix cracking, fiber breakage, and delamination models	Peak force, damage area, absorbed energy	ABAQUS	[38]

High velocity impact	Chang-Chang criteria	HVI	Hou criteria	Residual velocity and ballistic limit	ABAQUS/Explicit	39
Kevlar® fabric and polypropylene (PP) matrix	non-linear anisotropic constitutive	HVI	multi-failure modes under ballistic impact	Residual velocity, Area of damage and ballistic limit	ANSYS AUTODYN v. 14.0	40
Kevlar® Reinforced Thermoplastic	Hydrocode model with MAg-PP	HVI	Multi-failure modes including matrix cracking, delamination, and fiber failure	Ballistic limit, damage patterns	ANSYS AUTODYN	41
Carbon Fiber Reinforced Polymer (CFRP)	Mesoscale model	HVI	Delamination and fiber failure models	Ballistic limits, deflections	LS-DYNA	42
Armour Grade Steel	Elastic-plastic model	HVI	Shear plugging and delamination	Residual velocities, damage area	LS-DYNA	43

Combat Helmet (Aramid Composite)	Composite damage model	HVI	Fiber-matrix shear out, fiber breakage, Delamination and fiber failure	Back face deformation (BFD), ballistic limits, Residual velocities	ANSYS/LS-DYNA	44

Reference:

1. Thiruppukuzhi, S. V., and Sun, C.T. (1998). Testing and modeling high strain rate behavior of polymeric composites. Compos B Eng *29*, 535–546. https://doi.org/10.1016/S1359-8368(98)00009-2.
2. Jia, S., Wang, F., Zhou, J., Jiang, Z., and Xu, B. (2021). Study on the mechanical performances of carbon fiber/epoxy composite material subjected to dynamical compression and high temperature loads. Compos Struct *258*, 113421. https://doi.org/10.1016/j.compstruct.2020.113421.
3. Qian, X., Wang, H., Zhang, D., and Wen, G. (2016). High strain rate out-of-plane compression properties of aramid fabric reinforced polyamide composite. Polym Test *53*, 314–322. https://doi.org/10.1016/j.polymertesting.2016.06.006.
4. Bandaru, A.K., Chouhan, H., and Bhatnagar, N. (2020). High strain rate compression testing of intra-ply and inter-ply hybrid thermoplastic composites reinforced with Kevlar/basalt fibers. Polym Test *84*, 106407. https://doi.org/10.1016/j.polymertesting.2020.106407.
5. Arbaoui, J., Tarfaoui, M., and El Malki Alaoui, A. (2016). Mechanical behavior and damage kinetics of woven E-glass/vinylester laminate composites under high strain rate dynamic compressive loading: Experimental and numerical investigation. Int J Impact Eng *87*, 44–54. https://doi.org/10.1016/j.ijimpeng.2015.06.026.
6. Massaq, A., Rusinek, A., Klosak, M., Bahi, S., and Arias, A. (2019). Strain rate effect on the mechanical behavior of polyamide composites under compression loading. Compos Struct *214*, 114–122. https://doi.org/10.1016/j.compstruct.2019.01.101.

7. Zhang, Y., Liu, T., and Xu, Z. (2019). Dynamic response of hybrid carbon fibre laminate beams under ballistic impact. Compos Struct *210*, 409–420. https://doi.org/10.1016/j.compstruct.2018.11.049.

8. Gowda, D., Mahesh, V., Mahesh, V., and Ravishankar, K. (2024). Experimentation on dynamic compressive response of bio-inspired helicoidal structured Basalt/Hemp/polyurethane rubber sandwich composites. Mater Today Commun *38*, 108343. https://doi.org/10.1016/j.mtcomm.2024.108343.

9. Shaker, K., Jabbar, A., Karahan, M., Karahan, N., and Nawab, Y. (2017). Study of dynamic compressive behaviour of aramid and ultrahigh molecular weight polyethylene composites using Split Hopkinson Pressure Bar. J Compos Mater *51*, 81–94. https://doi.org/10.1177/0021998316635241.

10. Kim, W., Argento, A., Lee, E., Flanigan, C., Houston, D., Harris, A., and Mielewski, D.F. (2012). High strain-rate behavior of natural fiber-reinforced polymer composites. J Compos Mater *46*, 1051–1065. https://doi.org/10.1177/0021998311414946.

11. Paliwal, I., Ramji, M., and Khaderi, S.N. (2023). Experimental characterization of CFRP single lap joints under tension at various loading rates. Compos Part A Appl Sci Manuf *173*, 107636. https://doi.org/10.1016/j.compositesa.2023.107636.

12. Yan, K., Jiang, Z., Tang, J., Xie, X., and Suo, T. (2024). Experimental and numerical study on the loading rate dependent tensile behavior of carbon fiber/epoxy interface. Compos B Eng *284*, 111732. https://doi.org/10.1016/j.compositesb.2024.111732.

13. Omar, M.F., Md Akil, H., Ahmad, Z.A., Mazuki, A.A.M., and Yokoyama, T. (2010). Dynamic properties of pultruded natural fibre reinforced composites using Split Hopkinson Pressure Bar technique. Mater Des *31*, 4209–4218. https://doi.org/10.1016/j.matdes.2010.04.036.

14. Mengal, A.N., Karuppanan, S., and Ovinis, M. (2017). In–plane shear properties of basalt–carbon/epoxy hybrid composite laminates. Materwiss Werksttech *48*, 261–266. https://doi.org/10.1002/mawe.201600770.

15. Weng, F., Fang, Y., Ren, M., Sun, J., and Feng, L. (2021). Effect of high strain rate on shear properties of carbon fiber reinforced composites. Compos Sci Technol *203*, 108599. https://doi.org/10.1016/j.compscitech.2020.108599.

16. Schoeppner, G.A., and Abrate, S. (2000). Delamination threshold loads for low velocity impact on composite laminates. Compos Part A Appl Sci Manuf *31*, 903–915. https://doi.org/10.1016/S1359-835X(00)00061-0.

17. Faggiani, A., and Falzon, B.G. (2010). Predicting low-velocity impact damage on a stiffened composite panel. Compos Part A Appl Sci Manuf *41*, 737–749. https://doi.org/10.1016/j.compositesa.2010.02.005.

18. Sun, X.C., and Hallett, S.R. (2017). Barely visible impact damage in scaled composite laminates: Experiments and numerical simulations. Int J Impact Eng *109*, 178–195. https://doi.org/10.1016/j.ijimpeng.2017.06.008.

19. English, S.A., Briggs, T.M., and Nelson, S.M. (2016). Quantitative validation of carbon-fiber laminate low velocity impact simulations. Compos Struct *135*, 250–261. https://doi.org/10.1016/j.compstruct.2015.09.033.

20. Ginzburg, D., Pinto, F., Iervolino, O., and Meo, M. (2017). Damage tolerance of bio-inspired helicoidal composites under low velocity impact. Compos Struct *161*, 187–203. https://doi.org/10.1016/j.compstruct.2016.10.097.

21. Li, X., Ma, D., Liu, H., Tan, W., Gong, X., Zhang, C., and Li, Y. (2019). Assessment of failure criteria and damage evolution methods for composite laminates under low-velocity impact. Compos Struct *207*, 727–739. https://doi.org/10.1016/j.compstruct.2018.09.093.

22. Muñoz, R., Seltzer, R., Sket, F., González, C., and Llorca, J. (2022). Influence of hybridisation on energy absorption of 3D woven composites under low-velocity impact loading. Modelling and experimental validation. Int J Impact Eng *165*, 104229. https://doi.org/10.1016/j.ijimpeng.2022.104229.

23. Tuo, H., Lu, Z., Ma, X., Xing, J., and Zhang, C. (2019). Damage and failure mechanism of thin composite laminates under low-velocity impact and compression-after-impact loading conditions. Compos B Eng *163*, 642–654. https://doi.org/10.1016/j.compositesb.2019.01.006.

24. Antonucci, V., Caputo, F., Ferraro, P., Langella, A., Lopresto, V., Pagliarulo, V., Ricciardi, M.R., Riccio, A., and Toscano, C. (2016). Low velocity impact response of carbon fiber laminates fabricated by pulsed infusion: A review of damage investigation and semi-empirical models validation. Progress in Aerospace Sciences *81*, 26–40. https://doi.org/10.1016/j.paerosci.2015.11.002.

25. Katunin, A., Wronkowicz-Katunin, A., Danek, W., and Wyleżoł, M. (2021). Modeling of a realistic barely visible impact damage in composite structures based on NDT techniques and numerical simulations. Compos Struct *267*, 113889. https://doi.org/10.1016/j.compstruct.2021.113889.

26. Ismail, M.F., Sultan, M.T.H., Hamdan, A., Shah, A.U.M., and Jawaid, M. (2019). Low velocity impact behaviour and post-impact characteristics of kenaf/glass hybrid composites with various weight ratios. Journal of Materials Research and Technology *8*, 2662–2673. https://doi.org/10.1016/j.jmrt.2019.04.005.

27. Ismail, A.E., Zainulabidin, M.H. Bin, Roslan, M.N., Mohd Tobi, A.L., and Muhd Nor, N.H. (2013). Effect of Velocity on the Impact Resistance of Woven Jute Fiber Reinforced Composites. Applied Mechanics and Materials *465–466*, 1277–1281. https://doi.org/10.4028/www.scientific.net/AMM.465-466.1277.

28. Jusoh, M.S.B.M., Ahmad, H.A.B.I., and Yahya, M.Y. Bin (2017). Indentation and low velocity impact properties of woven E-glass hybridization with basalt, jute and flax toughened epoxy composites. In 2017 3rd International Conference on Power Generation Systems and Renewable Energy Technologies (PGSRET) (IEEE), pp. 164–168. https://doi.org/10.1109/PGSRET.2017.8251821.

29. Zuccarello, B., Militello, C., and Bongiorno, F. (2023). Environmental aging effects on high-performance biocomposites reinforced by sisal fibers. Polym Degrad Stab *211*, 110319. https://doi.org/10.1016/j.polymdegradstab.2023.110319.

30. Ahmed, K.S., Vijayarangan, S., and Kumar, A. (2007). Low Velocity Impact Damage Characterization of Woven Jute—Glass Fabric Reinforced Isothalic Polyester Hybrid Composites. Journal of Reinforced Plastics and Composites *26*, 959–976. https://doi.org/10.1177/0731684407079414.

31. Jalón, E., Hoang, T., Rubio-López, A., and Santiuste, C. (2018). Analysis of low-velocity impact on flax/PLA composites using a strain rate sensitive model. Compos Struct *202*, 511–517. https://doi.org/10.1016/j.compstruct.2018.02.080.

32. Sasso, M., Mancini, E., Dhaliwal, G.S., Newaz, G.M., and Amodio, D. (2019). Investigation of the mechanical behavior of CARALL FML at high strain rate. Compos Struct *222*, 110922. https://doi.org/10.1016/j.compstruct.2019.110922.

33. Asaee, Z., and Taheri, F. (2016). Experimental and numerical investigation into the influence of stacking sequence on the low-velocity impact response of new 3D FMLs. Compos Struct *140*, 136–146. https://doi.org/10.1016/j.compstruct.2015.12.015.

34. Jiang, H., Ren, Y., Liu, Z., Zhang, S., and Lin, Z. (2019). Low-velocity impact resistance behaviors of bio-inspired helicoidal composite laminates with non-linear rotation angle based layups. Compos Struct *214*, 463–475. https://doi.org/10.1016/j.compstruct.2019.02.034.

35. Lyu, Q., Wang, B., Zhao, Z., and Guo, Z. (2022). Damage and failure analysis of hybrid laminates with different ply-stacking sequences under low-velocity impact and post-impact compression. Thin-Walled Structures *180*, 109743. https://doi.org/10.1016/j.tws.2022.109743.

36. Lopes, C.S., Camanho, P.P., Gürdal, Z., Maimí, P., and González, E.V. (2009). Low-velocity impact damage on dispersed stacking sequence laminates. Part II: Numerical simulations. Compos Sci Technol *69*, 937–947. https://doi.org/10.1016/j.compscitech.2009.02.015.

37. Chen, D., Luo, Q., Meng, M., Li, Q., and Sun, G. (2019). Low velocity impact behavior of interlayer hybrid composite laminates with carbon/glass/basalt fibres. Compos B Eng *176*, 107191. https://doi.org/10.1016/j.compositesb.2019.107191.

38. Bandaru, A.K., Patel, S., Ahmad, S., and Bhatnagar, N. (2018). An experimental and numerical investigation on the low velocity impact response of thermoplastic hybrid composites. J Compos Mater *52*, 877–889. https://doi.org/10.1177/0021998317714043.

39. Peinado, J., Jiao-Wang, L., Olmedo, Á., and Santiuste, C. (2022). Influence of stacking sequence on the impact behaviour of UHMWPE soft armor panels. Compos Struct *286*, 115365. https://doi.org/10.1016/j.compstruct.2022.115365.

40. Bandaru, A.K., Chavan, V. V., Ahmad, S., Alagirusamy, R., and Bhatnagar, N. (2016). Ballistic impact response of Kevlar® reinforced thermoplastic composite armors. Int J Impact Eng *89*, 1–13. https://doi.org/10.1016/j.ijimpeng.2015.10.014.

41. Bandaru, A.K., Vetiyatil, L., and Ahmad, S. (2015). The effect of hybridization on the ballistic impact behavior of hybrid composite armors. Compos B Eng *76*, 300–319. https://doi.org/10.1016/j.compositesb.2015.03.012.

42. Chocron, S., Carpenter, A.J., Scott, N.L., Bigger, R.P., and Warren, K. (2019). Impact on carbon fiber composite: Ballistic tests, material tests, and computer simulations. Int J Impact Eng *131*, 39–56. https://doi.org/10.1016/j.ijimpeng.2019.05.002.

43. Dong, Y., Yang, L., Jin, Z., and Wu, L. (2022). Experimental and numerical analysis of ballistic impact response of fiber-reinforced composite/metal composite target. Compos Struct *294*, 115776. https://doi.org/10.1016/j.compstruct.2022.115776.

44. Rodríguez-Millán, M., Ito, T., Loya, J.A., Olmedo, A., and Miguélez, M.H. (2016). Development of numerical model for ballistic resistance evaluation of combat helmet and experimental validation. Mater Des *110*, 391–403. https://doi.org/10.1016/j.matdes.2016.08.015.
